# Decreasing the Uncertainty in the Comparison of Molecular Fingerprints of Organic Aerosols with H/D Exchange Mass Spectrometry

**DOI:** 10.1021/acs.est.4c06749

**Published:** 2024-11-08

**Authors:** Alexander Zherebker, Oliver Babcock, Diana L. Pereira, Sara D’Aronco, Daniele Filippi, Lidia Soldà, Vincent Michoud, Aline Gratien, Manuela Cirtog, Christopher Cantrell, Paola Formenti, Chiara Giorio

**Affiliations:** 1 Yusuf Hamied Department of Chemistry, University of Cambridge, Cambridge CB2 1EW, United Kingdom; 2 Université Paris Cité and Université Paris-Est Créteil, CNRS, LISA, Paris F-75013, France; 3 Department of Chemical Sciences, Università degli Studi di Padova, 35131 Padova, Italy; 4 Université Paris Est Creteil and Université Paris Cité, CNRS, LISA, Créteil F-94010, France

**Keywords:** high-resolution mass spectrometry, structural descriptor, labile protons, isomers, KMD series, nontarget analysis, chemical pairs

## Abstract

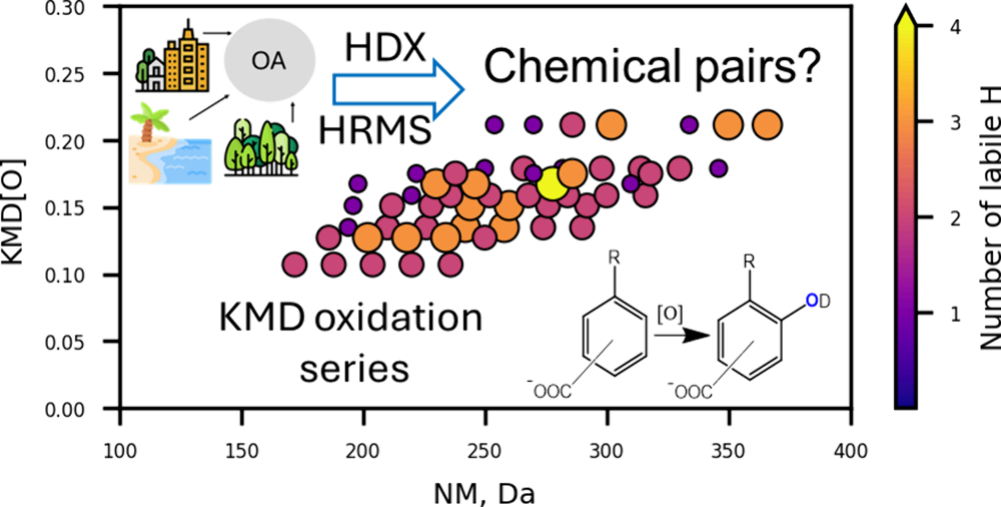

High-resolution mass spectrometry (HRMS) has become an indispensable tool in the characterization of organic aerosols (OA) providing information on air quality, health assessment, climate trends, reactions, and source apportionment. Spectra-derived lists of formulas and their relative abundances are used to compare ambient OA from different sources or to monitor secondary OA formation under controlled laboratory conditions in smog chamber experiments. Various techniques are implemented to visualize common and unique features, series of precursors, and products. The disadvantage of this conventional approach is in associating elemental compositions to specific compounds, while due to several analytical limitations, the structural information remains hidden. We argue that some of the conclusions derived from this data analysis can be misleading. In this study, we applied in-ESI source H/D exchange (HDX), which facilitated enumeration of labile protons in molecules behind elemental compositions of OA. We applied this technique to compare OA from three different locations representing urban, forest, and marine environments and to examine tentative chemical information derived from Kendrick mass defect (KMD) series analysis. Significant discrepancies were found between numbers of labile protons in protogenic functional groups and the stoichiometry of chemical reactions, which are associated with KMD series. Only a portion of chemical pairs matched target stoichiometries, which highlights the existing limitations in environmental applications of conventional formula-based HRMS data interpretation strategies.

## Abstract 2

Analysis of mass spectrometry data is based on strong assumptions. Application of H/D exchange enabled to distinguish correct and false precursor−product pairs in organic aerosols.

## Introduction

Various organic molecules can make up to 70% of aerosol particles’ mass.^1^ While the toxicity effect of particulate-bound metals is relatively well understood,^2^ organic constituents can also directly impact health while also playing a key role in absorbing and scattering solar and terrestrial radiation and interacting with clouds, thereby affecting the Earth’s climate.^3,4^ In this regard, high-resolution mass spectrometry (HRMS) has become an indispensable tool for analyzing organic aerosols (OA) providing information about their elemental composition.^5−7^ Well-annotated spectra are routinely obtained to trace biomass burning markers,^8^ to investigate rural, urban, and marine aerosols,^9−11^ and to assess chemical mechanisms of secondary OA formation under controlled laboratory conditions in smog chamber experiments.^7,12,13^ Thousands of oxidized organic compounds are found in a single HRMS spectrum, including CHO, CHON, CHOS, and CHONS species. Combination with liquid chromatography empowers HRMS, providing also information about compound polarity and possible isomeric complexity.^14−16^ By analyzing samples collected from various sources, it is possible to find conservative and variable components of OA magnifying differences between atmospheric processes.^17−19^ Alternatively, source apportionment can be estimated for a single set of samples.^9,11,20^ For example, applying HRMS to study the water-soluble organic carbon (WSOC) fraction of OA collected from three different locations in the Atlantic Ocean allowed to identify which site represents almost pure marine aerosols and which was substantially affected by terrestrial emissions from the coast.^21^

The immense data size provided by HRMS requires some formalization. Several formula-derived features were developed to compare the chemistry of different components, among which the aromaticity equivalent^22^ and the nominal oxidation state of carbon^23^ are those utilized the most. Additionally, several visualization techniques are used to depict and explore the molecular ensembles of OA samples. The Van Krevelen (VK) diagram shows the interconnection between oxidation and condensation of OA components.^24^ The double bond equivalent (DBE) plotted versus number of carbon or molecular mass shows the contribution of low- and high-molecular weight unsaturated and saturated compounds.^25^ The Kendrick diagram facilitates visualization of homologue series differing by the specific small moiety.^26,27^ Additionally, Kendrick mass defect is used to highlight molecular components, which can constitute precursors/products chemical pairs because their specific differences in elemental composition fit reaction stoichiometries.^6,28,29^ For example, matching all differences to metabolomic pathways can be applied to estimate the number of chemical transformations between components of a complex mixture.^30,31^

Despite the doubtful advantages of current HRMS approaches, one can argue that the elemental composition level is insufficient for a confident identification.^32^ Consequently, associating common formulas with common molecules can be misleading. It was shown that operating with Kendrick diagrams can result in an overestimation of functional groups^33^ while formula-derived features may lead to an inaccurate estimation of compound aromaticity.^34^ Tandem mass spectrometry may provide structural information enabling higher confidence of tentative structural attribution.^15^ However, in the case of aged OA, reliable spectral annotation can be performed only to compounds with existent standards or to a limited number of ions due to absence of fragmentation libraries and possible structural rearrangements after bond cleavage.^35,36^ Therefore, integral information on functional groups provided by FTIR^37,38^ and NMR^39^ spectroscopy for the bulk OA samples is missing for individual components. To partially account for the abovementioned issues, in this study, we combined conventional direct injection HRMS analysis of aerosol samples with in-source hydrogen/deuterium exchange (HDX),^40,41^ which allows obtaining information on the number of functional groups in individual carriers in addition to simple elemental composition. This data was used to provide tentative chemical information on VK diagrams and to examine the ability of Kendrick diagrams to reflect precursor/product chemical pairs. Experiments have been performed with OA samples collected in urban, forest, and marine environments for which substantial structural variability was expected.

## Materials and Methods

### Solvents and Reagents

Solvents, consumables, and other reagents used in this study were all commercially available. Methanol of LC-MS grade (Fisher Scientific) was used for the sample preparation and analysis. Ultrapure water (18.2 MΩ) was prepared using a Merck Millipore Advantage A10 Water Purification System. Solution of 0.1% formic acid (FA) was prepared from LC-MS grade ≥99% FA (HiPerSolv CHROMANORM). HDX experiments were performed with 99.8% D_2_O (Thermo Scientific). Bond Elut PPL cartridges (50 mg, 1 mL) were used for solid-phase extraction (SPE).

### OA Samples

Three urban background aerosols (PM_2.5_) were collected during 24 h in winter 2019 in Padua (Italy), as described elsewhere.^42^ Eight forest samples (PM_1_) were collected both at night (22:00–6:00, local time) and at daytime (6:00–22:00, local time) as part of the Atmospheric Chemistry of the Suburban Forest (ACROSS) project in summer 2022 in the Rambouillet Forest (ca. 50 km from Paris).^43,44^ Eight marine aerosol samples as total suspended particles (TSP) were collected as part of the Aerosols, Radiation and Clouds in Southern Africa (AEROCLO-sA) project at the coastal site of Henties Bay (Namibia) in September 2017 during different time periods (from 7 to 23 h), as described elsewhere.^45^ Before extraction, filters were stored in prebaked and sealed aluminum foils at −20 °C. Forest aerosols were extracted with methanol, while urban and marine samples were extracted with water followed by SPE and methanol elution. Methanol was chosen to extract less polar compounds contributing to forest OA, while water followed by SPE was applied to remove salts that are present in urban and marine OA. To examine the impact of the solvent on the molecular composition of OA, a section of filter from the urban environment was extracted with methanol similarly to the extraction method used for forest samples. The mass of particles deposited on filters varied from 0.4 to 3 mg estimated by using reported PM concentrations and collection times. Estimation of particles’ mass may contribute to the uncertainty of comparison of the samples. Detailed extraction procedures can be found in the Supporting Information. Urban and marine OA were analyzed the next day after extraction, and forest OA were stored in the freezer for 2 months.

### HRMS Analysis and HDX Experiments

High-resolution mass spectra were obtained by using an LTQ Orbitrap mass spectrometer (Thermo Scientific, Bremen, Germany) equipped with a heated electrospray source (HESI) operating in negative ion mode. Sample solutions were injected using a syringe pump with a flow rate of 6 μL min^–1^ and a needle voltage of −3.3 kV. For rapid dissolution and to ensure HDX, capillary temperature and sheath gas values were set to 300 °C and 2 au, respectively. For each parent sample, 100 scans were accumulated in triplicates for two scan ranges (50–500 and 150–700 *m*/*z*) with a nominal resolution of 100,000. Two mass segments were used to facilitate efficient detection of low- and high-molecular weight compounds.^46^ Three types of blanks (solvent, laboratory blank, and field filter blank from the same batch of filters) were analyzed in the same manner. For HDX experiments, 150 μL of D_2_O was placed on a thin copper plate in the HESI source below the needle, which facilitates high exchange efficiency even for complex mixtures, as it was described previously for natural organic matter.^47,48^ Spectra were acquired in the same manner but with accumulation of 200 scans without replicates.

### Data Treatment

Raw HRMS data were treated using open-source software and lab-written Python scripts. Visualization of data was performed with Python library Matplotlib (https://matplotlib.org/). Statistical analysis was performed with Python libraries NumPy, Pandas, and Seaborn. All *.raw files were converted to *.mzML format using msconvert with the vendor-recommended peak-picking algorithm (https://proteowizard.sourceforge.io/). Peak lists were extracted using the Python script based on the Pyteomics library (https://pyteomics.readthedocs.io/en/latest/). Formula assignment for all replicates of parent samples was conducted with the Python script based on the open-source NOMspectra Python library^49^ (https://NOMspectra.readthedocs.io/en/latest/) with a denoising step adapted from Zielinski et al.^50^ using the following constraints: *O*/*C* ratio ≤2, 0.3 < *H*/*C* ratio <2.5; element counts [1 < *C* ≤ 60, 2 < *H* ≤ 100, 0 < *O* ≤ 60, *N* ≤ 2, *S* ≤ 1]; *z* = −1; and mass accuracy window <0.5 ppm after built-in internal calibration based on the construction of the probability density of assignments.^51^ Only formulas found in all three replicates were retained, and peaks presented in any of the blank analysis were removed from the final assignment to account for possible contaminations during extraction and analysis. All the molecular formulas found in the two mass ranges were combined; if molecular formulas were found in both ranges, the one having the higher intensity was retained, and the signal intensity was then normalized to the highest peak.

On the next step, molecular assignments presented in at least one sample of each OA type were concatenated in a new source-representative table giving aggregated data for urban, forest, and marine OA. This step aimed to decrease the uncertainty of qualitative source comparison raised from the unknown mass balance. Furthermore, molecular formulas from each table were divided into two subsets: common formulas, which were found in all sites, and unique formulas, which were present only in one site. For each formula, the constrained aromaticity index (AI_con_) was calculated, which served a good proxy for aromaticity of components of natural organic matter.^34^ AI_con_ was further used to divide formulas into aromaticity-based classes.^52^ Constraints for AI_con_-based classes are provided in the Supporting Information.

Data treatment for the HDX spectra was conducted in the same manner, skipping the formula assignment step. The number of labile protons was automatically assigned using the Python script adapting the previously reported algorithm^47^ with heuristic rules adapted from Zherebker et al.^34^ Essentially, the algorithm juxtaposes mass spectra before and after HDX and searches for the peak series with Δ*m*/*z* = 1.00628 (with mass tolerance of 1 × 10^–4^ Da) corresponding to the HDX reaction. For each of the sample, HDX series were calculated and the minimum number of labile protons was chosen to combine with each source-related formula list. The Python code for HDX calculations and test data are provided at https://github.com/AZherebker/Calculation-of-HDX-series/

## 3 Results and Discussion

### 3.1 Molecular Fingerprints of OA Samples Derived from HRMS Analysis

In total, three, eight, and eight formula lists were obtained for urban, forest, and marine sources, respectively, with a number of formulas varying from 500 to 2700. The number and relative abundance of CHON assignments were high in all cases (Figure S1), which is consistent with a previous study of OA.^53^ Despite the number of CHOS formulas being low, for marine and forest samples, their intensity contribution reached 30 and 40%, respectively, while in urban samples, the contribution was below 10%. High contribution of CHOS compounds in forest aerosols was reported previously, and it is related to both biogenic and anthropogenic origins.^18,19^ Pairwise cosine and Jaccard similarity indices were calculated to compare samples, and the resulting heatmaps are presented in Figure S2A,B. Three distinct clusters can be seen, which were in accordance with the sampling sites with higher variability between samples in marine OA. This clearly indicates the substantial difference between samples’ compositions. It is worth noting that the number of pairwise shared formulas was dependent on the total number of assignments. Consequently, for Namibia samples, the pairwise intersection varied from 45 to 814 for samples with the highest number of assignments (Figure S3B). The number of shared formulas between urban and forest samples varied from 572 to 1230. Although the estimated concentrations of organic carbon were sufficient for the analysis, the low number of assignments in the case of marine OA samples may correspond to lower particle mass, fractional size,^54^ and ionization efficiency of their components in negative electrospray biased toward acidic and oxidized compounds.^55^ Taking the difference in the number of assignments, it was of interest to assess how the distribution of only shared formulas correlated. Figure S3A shows the correlation for the intersections between the samples. The urban samples were still distinct, while forest and marine samples showed a significant correlation, which indicates the presence of conservative components of OA that undergo similar transformations at different environments or the possible long-range transport of secondary marine OA, which was detected in Paris.^56^ High abundances of CHOS formulas indicated the same mechanism of photooxidative formation of organosulfates in these two areas.^13,57^

**Figure 1:**
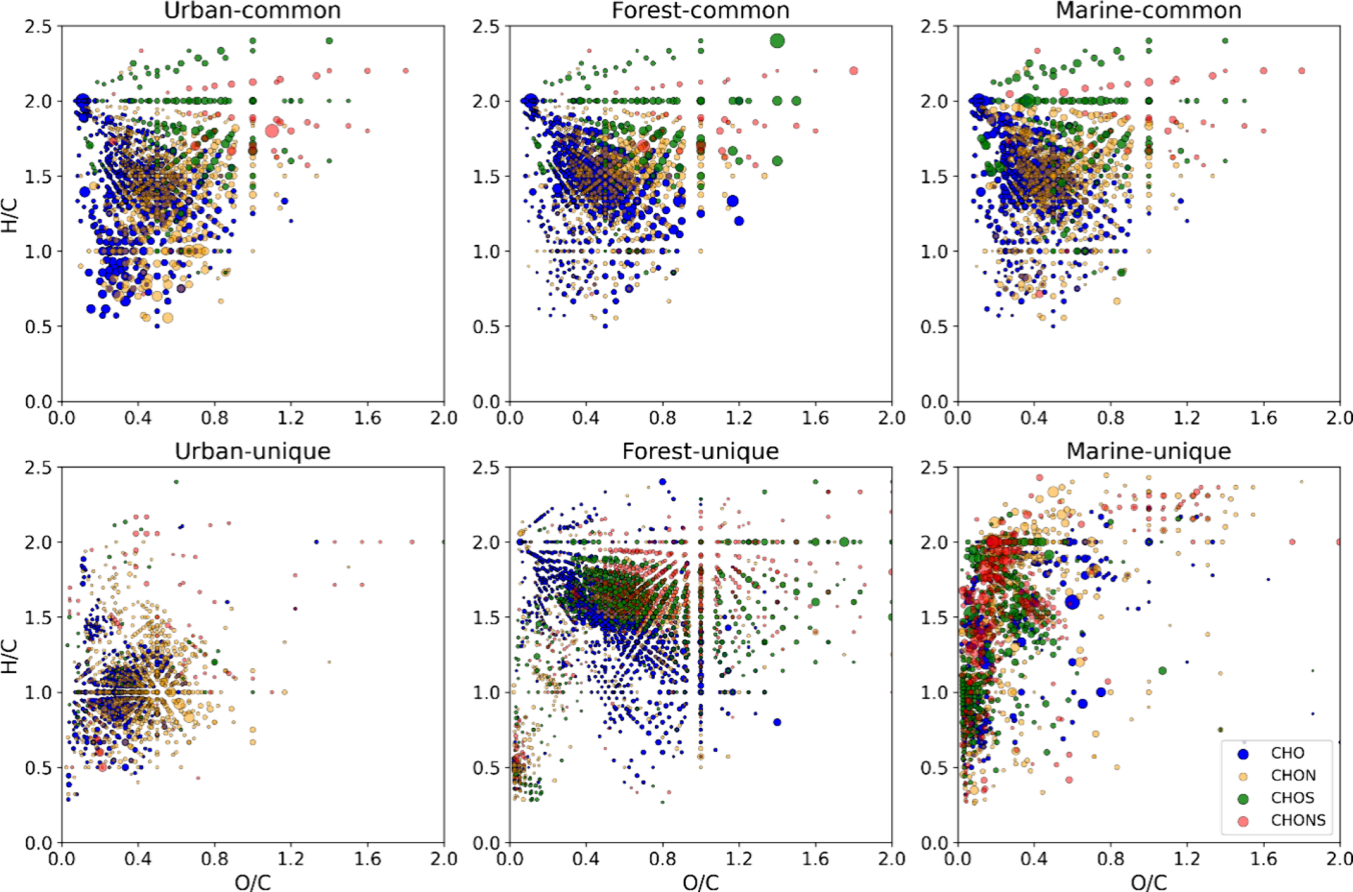
VK diagrams show common and unique molecular features for each OA type. The size of the dots reflects the relative intensity of each component.

Samples' fingerprints plotted as VK diagrams are presented in Figure S4. VKs show significant heterogeneity of the samples. Urban samples were highly populated with low oxidized highly unsaturated compounds with *H*/*C* < 1.5 but also included highly oxidized saturated compounds. Forest OA samples were enriched with unsaturated oxidized components as well as highly oxidized CHOS species. Marine CHO components occupied the same area on VK diagrams as in the case of forest samples. CHOS formulas for marine samples showed a distinct pattern with high contribution of low oxidized S-containing components. It was of interest to identify common and unique molecular features of OA sources under study. The results are presented in Figure 1 as VK diagrams and in Figure S5 as intensity contribution of different AI_con_-based classes for common and unique components. Common formulas occupied a wide area on VK diagrams covering both highly saturated compounds with *H*/*C* > 1.5 and compounds with *H*/*C* < 1 and AI_con_ > 0.5. All sources included highly oxygenated saturated S-containing compounds. Consideration of unique formulas showed almost a puzzle-like site-specific area on VK diagrams. Marine OA was explained mostly by reduced compounds with different degrees of unsaturation. Urban OA occupied an area of highly unsaturated oxidized compounds.^58^ Forest OA was distinct by the highest population of oxidized saturated compounds including CHO, CHOS, and CHON species. Despite forest samples being extracted with methanol, while urban and marine samples were extracted with water, the differences observed between the sites cannot be attributed to the different extraction protocol. Comparison of water and methanol extracts for urban OA (Figure S6) showed similar molecular fingerprints with a slight increase in the proportion of components with higher AI_con_. This corroborates well with the literature reporting enrichment of methanol extract with aromatic compounds.^58^ Different particle sizes may also add to the variation in molecular compositions. In fact, it was shown that PM_1_ can be enriched with highly unsaturated compounds as compared to PM_2.5_ and total suspended particles while the latter is enriched in more saturated components.^54,59^ Yet, it is expected that the major portion of formulas is common for all fractions with the higher number of assignments for smaller fractions. Thus, the differences between studied OA likely correspond to variations in OA precursors and atmospheric variables such as solar irradiation, temperature, and humidity.^60^ For example, sesquiterpenes contribute significantly to forest OA due to accelerated oxidation rates.^61,62^ At the same time, higher abundance of aromatic components in urban OA corroborates with the previous reports showing their anthropogenic origin.^63^

The histograms for AI_con_-based classes (Figure S5) highlight the abovementioned differences semiquantitatively with some uncertainty due to differences in ionization efficiency of OA components. Marine OA were found to be enriched with saturated N-containing molecules and various aliphatic and low-oxidized unsaturated compounds, while urban OA were mostly represented by low-oxidized aromatic and highly oxidized unsaturated compounds. Forest OA partially included characteristics from both marine and urban sources: unique forest species were represented by reduced and oxidized saturated compounds, while dominant common species also included oxidized unsaturated compounds. Thus, the samples under study are composed of unique and common molecular features.

### 3.2 Distribution of Labile Protons in OA Samples

**Figure 2:**
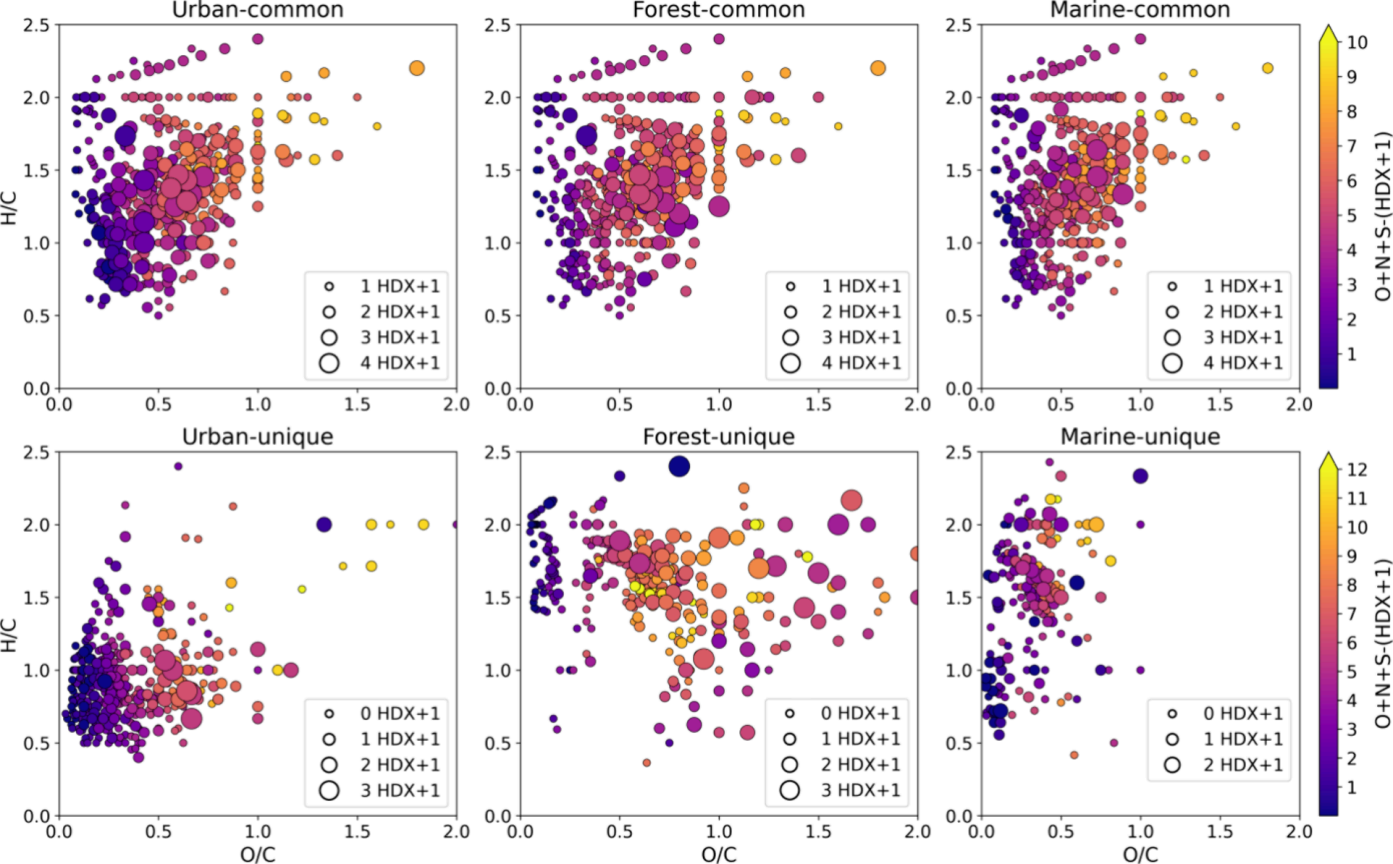
VK diagrams with the size-coded number of labile protons (HDX+1) and the color-coded number of residual heteroatoms (nonprotogenic heteroatoms) for common (upper panel) and unique (lower panel) molecular features for each OA source.

It was of interest to further assess functional groups of common and unique molecular features. The number of HDX varied from 0 to 3 giving a lower estimate for the labile protons in the range from 1 to 4 since one additional labile proton is responsible for the ionization, shown as HDX+1 in Figure 2. Despite that the actual number of labile protons can exceed the detected number, it was shown that the yield of in-ESI source HDX is high, and it facilitates exchange of all labile protons in small molecules such as amino acids and polyphenols.^64^ It is worth noting that in the mid pH values utilized in this study, the yield of HDX remains the same but may drop at pH above 10.^65^ Additionally, the traces of salts that may be present in methanol extracts and their scarce impact on HDX extent have been examined by the addition of up to 10% of (NH_4_)_2_SO_4_,^66^ as it is shown in Figure S7. To obtain comprehensive data for source-specific OA components, the information on the observed numbers of labile protons and the number of residual nonprotogenic heteroatoms was added to the VK diagrams. The resulting plots are depicted in Figure 2. Operating simultaneously with four features provides some clear benefits in characterization and comparison of OA samples with HDX HRMS. Considering common molecular assignments, we can see conservative and variating features between sources. Compounds with the same number of labile protons and, consequently, the same number of residual heteroatoms can be observed in various regions of the VK diagrams. Composition-specific distributions of labile protons for common formulas are shown in Figure S8. It should be noted that it was impossible to reliably determine HDX series for low-abundance OA components. Therefore, further discussion of chemical classes is relevant only for components with enumerated labile protons: 1629, 1581, and 1168 out of 2403, 3706, and 2725 formulas for the urban, forest, and marine OA, respectively.

It is seen that common unsaturated and aromatic components of urban OA contained up to four labile protons with a low content (ca. 3–4) of residual nonprotogenic heteroatoms. This was observed for all CHO, CHON, and CHOS species (Figure S8). This indicates the presence of hydroxylated aromatic rings, phenols, and nitrophenols, which can be of anthropogenic origin.^20^ Expectedly, the number of labile protons increased with the O/C ratio, which was also observed for the OA from other sources. Saturated reduced compounds (with low O/C ratio) were characterized by one or two labile protons and zero or one nonprotogenic heteroatom, indicating the presence of fatty acids and ruling out the presence of amino acids for which at least three labile protons are expected. The low number of labile protons for detected CHON components indicates the lack of amino groups, which are better detected in components ionizable in positive rather than negative ion mode in ESI.^67^ Saturated CHOS compounds also contained one or two labile protons but included up to seven nonprotogenic O and S atoms, which enables one to suggest that most of these compounds are sulfates, which accounts for four oxygen atoms and only one labile proton. Forest and marine OA components were characterized by the highest number of labile protons in oxidized species, including CHOS.^68^ Together, these data indicate that despite common molecular composition, organic constituents in OA of different origins may represent isomers with different sets of functional groups signaling different formation mechanisms and the role of anthropogenic emission.

Unique formulas highlight additional features of OA samples under study. Urban aerosols were represented by components that can be tentatively assigned to polyphenols with a lack of nonprotogenic groups. Marine OA included highly unsaturated (AI_con_ > 0.5) low-oxidized CHOS and CHON compounds, which likely represented substituted heterocycles such as thiophens.^68^ At the same time, saturated CHOS compounds contained one or two labile protons and up to three oxygen atoms, indicating the contribution of sulfonic acids. Oxidized species of forest OA were characterized by both high number of labile protons and nonprotogenic heteroatoms, pointing out complexity of their structures.

In some cases, HDX data can be used to constrain possible functional groups or to even determine them. For example, the carboxyl group contains two oxygen atoms providing only one proton; therefore, at least one residual oxygen atom is required for the presence of carboxyl groups. Table S3 shows numbers of CHO formulas from lipids (O ≥ 2, O/C < 0.3, and H/C ≥ 1.5), which can contain carboxyl groups. It is seen that most of the species are likely carboxylic acids. Yet, forest OA samples included three lipid-like components without residual oxygen atoms. These components could be falsely attributed to fatty acids, but they corresponded to polyatomic alcohols without carboxyl groups.

The oxidation state of sulfur atom can also be constrained for some CHOS components.^69^ Sulfate and sulfonic moieties include five and four heteroatoms, respectively, providing only a single exchangeable proton. Sulfoxide and sulfonyl lack protons but require two and and residual heteroatoms, respectively. The thiol group behaves the same way as the hydroxyl group contributing to one HDX per each sulfur atom. Table S4 shows the number of CHOS species with up to five oxygen atoms, which may include these types of S-containing moieties. Among detected components, 80–87% may correspond to sulfates and sulfonates. Thiol groups were predicted for forest and marine OA and were absent in the urban sample.

**Figure 3:**
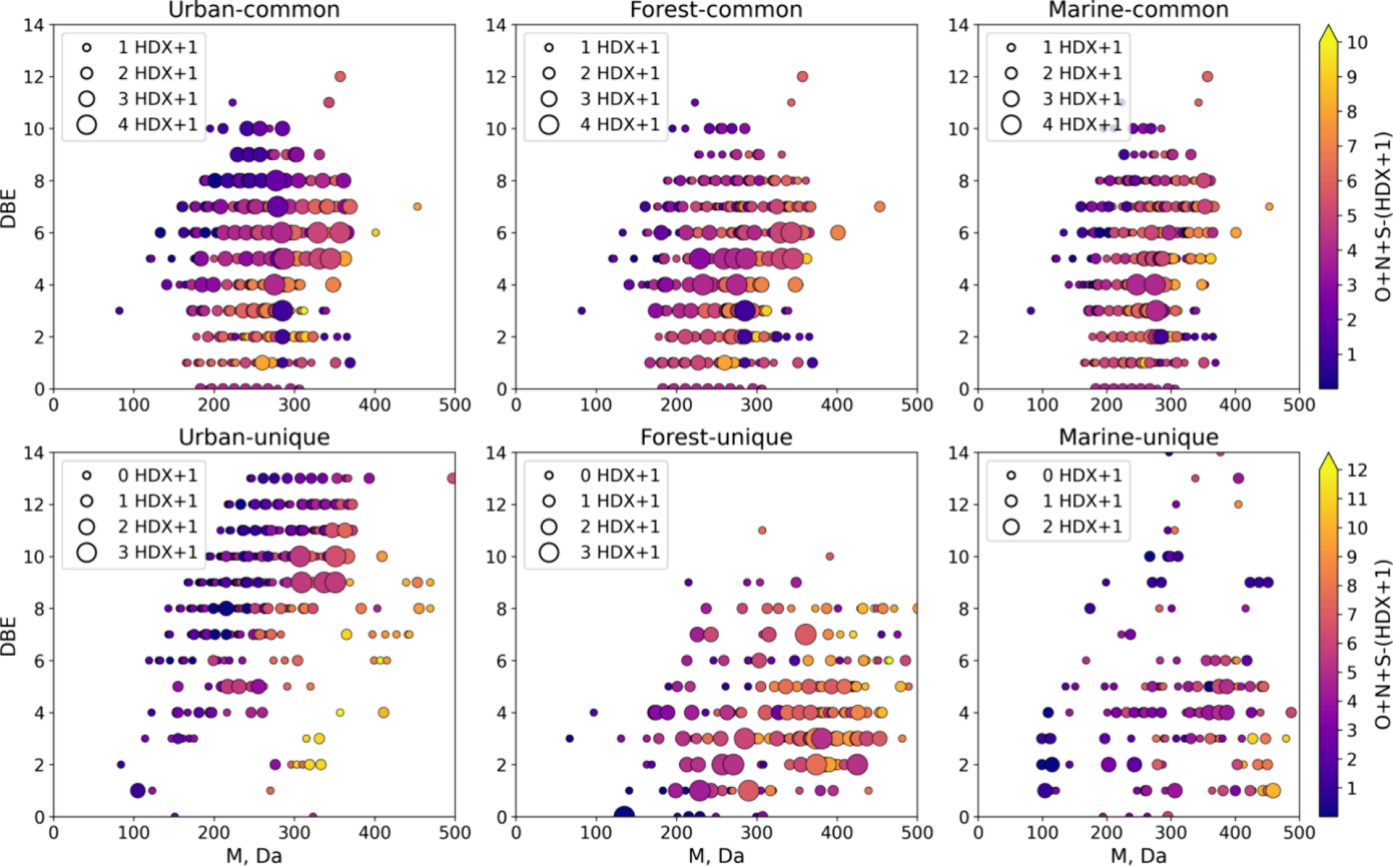
Double bond equivalent (DBE) versus molecular mass plots with the size-coded number of labile protons (HDX+1) and the color-coded number of residual heteroatoms (nonprotogenic heteroatoms) for common (upper panel) and unique (lower panel) molecular features for each OA source.

The DBE versus mass diagram enables us to better assess the contribution of protogenic groups to the compound’s structure. Figure 3 shows differences in the DBE series in three OA sources. Figures S9 and S10 depict such plots for CHO and CHOS components, separately. The heaviest unique molecules in urban OA are highly condensed with a DBE up to 14. Most of these molecules have a low number of both labile protons and heteroatoms. Unique forest OA compounds were characterized by low DBE values, high molecular weight, and high number of both labile protons and residual heteroatoms. The number of the latter increased with the mass forming series, with the same DBE values. This may indicate the formation of sulfates and sulfonates, which increases the mass without affecting the DBE. Unique marine OA components covered the whole mass range from 100 to 500 Da and were characterized mostly by low DBE values (<6). In low molecular weight compounds, labile protons and residual heteroatoms contributed similarly to OA components. CHOS compounds in which all heteroatoms were bonded to labile protons were observed in this region. At the same time, unlike forest OA, there was a lack of significant increase in the number of heteroatoms in high molecular weight compounds with DBE above 3 indicating that, within the DBE series, the increase in molecular mass should be related to the enlargement of the carbon skeleton rather than formation of sulfates or nitrates. The dealkylation processes have been discovered as one of the reaction pathways for aromatic compounds of OA.^70^ Depending on the functional groups of alkyl moieties, dealkylation may add, decrease, or retain the same number of labile protons. That is why there was a lack of a decreasing trend for marine DBE series. For saturated compounds with DBE below 3, the increase in molecular mass was accompanied by the increase in heteroatom content, indicating functionalization of aliphatic compounds in the atmosphere.

### 3.3 Tentative Assignment of Chemical Pairs Using KMD Series and HDX Data

**Figure 4:**
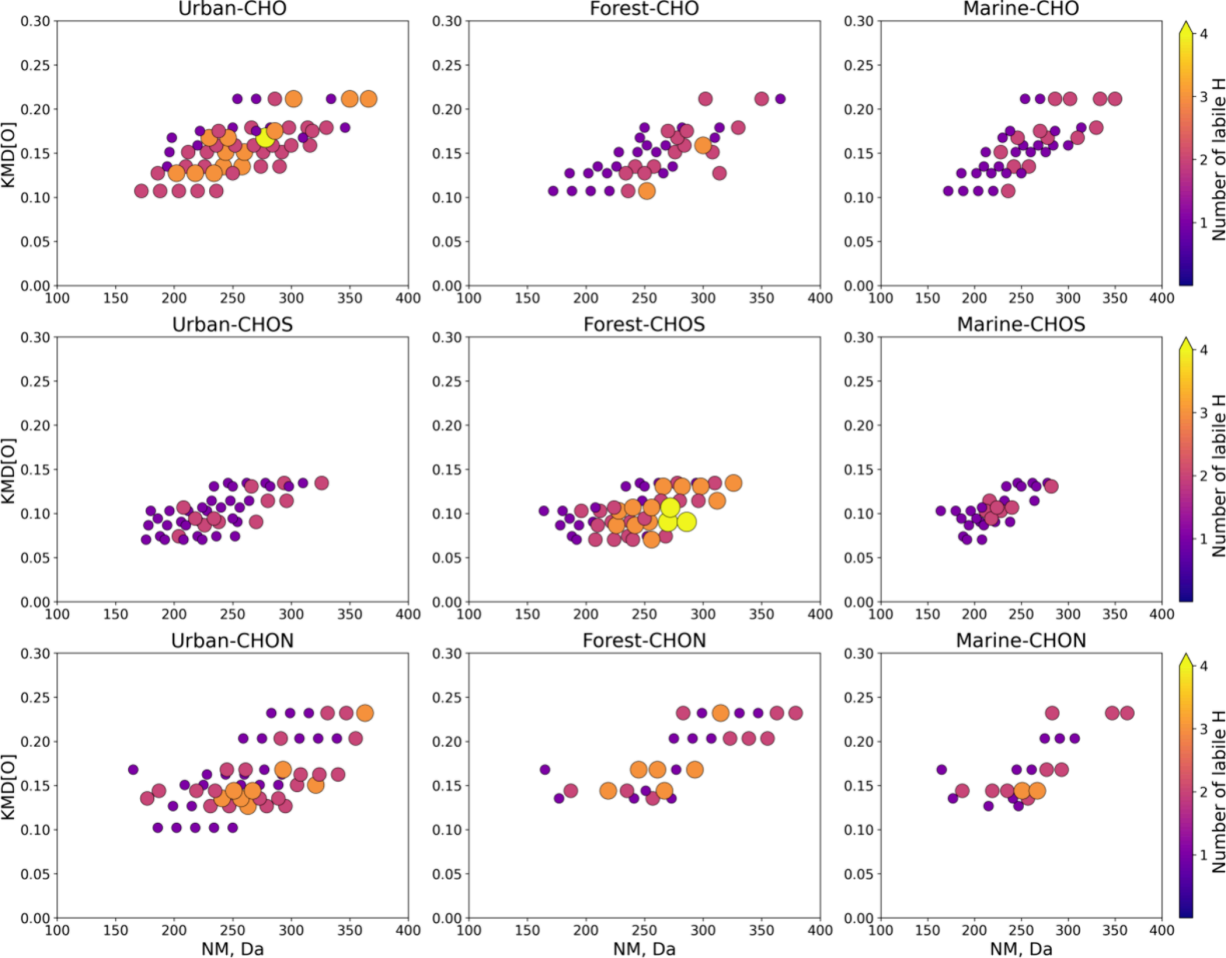
Selected KMD[O] series for CHO, CHON, and CHOS molecular components of OA from different sources with color- and size-coded numbers of labile protons.

Application of HDX enabled us to examine how accurate KMD diagrams are for describing chemical series of OA of different origin. Figure S11 depicts the KMD[CH_2_] diagram showing CH_2_ homologues for CHO and CHOS molecular series. It is clearly seen that the number of labile protons remains the same for almost each KMD[CH_2_] value, indicating that CH_2_ homologue series most likely represent the same chemical family or compounds with similar sets of functional groups. This also indicates that possible formation of acetals and esters by reaction of OA with methanol is minor in all cases.^71^ It should be highlighted that HDX provides information on the total number of labile protons rather than on specific functional groups; therefore, discussion on structural similarity can be conducted only on the tentative level.^32^ Oxygen series (O-series) are not clearly visualized on the KMD[O] diagram (Figure S12). The magnified series are shown in Figure 4 for CHO, CHON, and CHOS compounds. CHONS compounds had lower abundance, and they are not shown here.

In all cases, a long O-series can be observed, which is often interpreted as an indication of oxidation pathways connecting OA components. However, the HDX results highlight the speculative character of such discussions. We observed that within the O-series, the number of labile protons may remain the same or may increase or decrease regardless of the number of oxygen atoms. That is indicative of three types of chemical series in OA. The first type represents the actual chemical pairs of hydroxylation. We can see that in some cases, the next member of the O-series in fact included one more labile proton provided by the OH group. The second type represents molecules that differ exclusively by the number of oxygen atoms but have different structures, which were likely formed from different precursors or different reaction pathways and, therefore, cannot be interpreted as chemical pairs. The third type includes molecules that differ by the number of oxygen atoms but represent other chemical pairs. This can be visualized on the example of sulfonate and sulfate formation (structures a and b, respectively) from benzoic acid:^72^ while varying by one oxygen atom, the model CHOS structures represent two different chemical pairs with the same precursor.
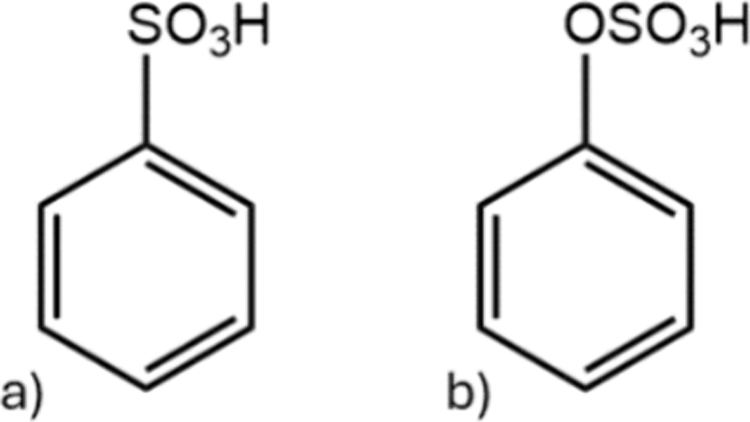


From the variety of possible chemical series, we focused on the interconversion of CHO compounds to organonitrates and CHO and CHON species to organosulfates due to simplicity of the stoichiometry of such transformations. Organonitrates may undergo hydrolysis in cloud droplets releasing one additional labile proton while formation of organosulfate or organosulfonate should not affect the number of labile protons.^29^ By comparing corresponding KMD pairs and results of HDX experiments, it was possible to distinguish between components that may represent actual chemical pairs or represent a second type of pairs similar to the O-series. The results are presented in Table 1.

**Table 1:** Numbers of Precursor–Product Pairs for Organonitrate Hydrolysis ([NO_2_(H_–1_)]), as well as Formation of Organosulfate and Organosulfonate with Sulfuric Acid ([SO_3_]) or SO_2_ ([SO_4_]) in OA from Different Sources Confirmed or Contradicted by HRMS, KMD, and HDX^a^

	**mass difference**		
OA source	**[SO**_**3**_**]**^b^	**[SO**_**4**_**]**^b^	**[NO**_**2**_**(H**_**–1**_**)]**^b^
marine	14/7	14/10	27/59
urban	10/32	15/14	64/162
forest	31/41	31/29	69/115

a The hydrolysis of organosulfate also matches the [SO_3_] mass difference.

b Numbers of HDX supported or disproved chemical pairs are divided by a slash.

For the R–ONO_2_ + H_2_O → R–OH + HNO_3_ series, we found significant discrepancies between KMD analysis and the HDX results. In the case of marine OA, only 27 out of 86 possible pairs showed the increase in the number of labile protons. Many more pairs were found for urban and forest aerosols, 64 and 69, respectively, which could be of both biogenic and anthropogenic origins. Formation of organosulfates or organosulfonates may be facilitated by the direct reaction of double-bond containing CHO and CHON species^73^ or by pseudoesterification with sulfuric acid in the atmosphere.^29^ Additionally, organosulfates may undergo hydrolysis in the clouds, resulting in the release of one OH group but without changing the number of labile groups. These reactions form SO_3_ and SO_4_ KMD series. Corresponding series were found in all OA, but ∼50% of them showed discrepancies with HDX data. Cumulatively, 28, 25, and 62 KMD pairs can be related to organosulfate formation reaction in marine, urban, and forest OA, respectively. It is worth noting that attribution of OA components to chemical pairs by HDX was mass independent, as it is shown in Figure S13.

## Environmental Implications

OA in the troposphere play an important ecological role and influence the Earth’s climate. Absorption of light, cloud formation properties, new particle formation, stability of greenhouse gas sinks, and impact on human health depend on the chemical structures of OA constituents. The closest level of molecular exploration can be achieved with HRMS, which covers a wide range of ionizable compounds providing semiquantitative information on their abundance when comparing samples from different environments. Due to the absence of analytical standards, the comparison currently relies on strong assumptions about the origin of organics in aerosols, tentative structures, and chemical processes. HRMS data are used for elucidation of common and unique molecular components and source apportionment leading to conclusions based on the aforementioned assumptions. In this study, we attempted to examine the chemical relevance of conventional data interpretation strategies and found substantial discrepancies with the experimental results including structural information. We found that while nontarget HRMS is capable of differentiating OA by their molecular fingerprints, formula-derived assumptions on structural similarity (e.g., same aromaticity or the presence of the same functional groups), mechanisms of their formation, origin, and reactivity should be avoided. The disagreement between the number of labile protons in individual species and their relationships derived from KMD analysis indicates the hidden complexity beyond the HRMS data. In our opinion in the current state, this complexity should prevent formula-based attribution of OA components to chemical structures, although it might be tempting in some cases. For example, laboratory experiments enabled to describe possible mechanisms of atmospheric synthesis of various organosulfates from biogenic precursors, such as isoprene, α-pinene, and β-pinene.^74^ The similar mechanism would be expected for samples under the current study. For example, in forest OA, we found the molecular composition of C_5_H_12_O_7_S, which possessed high abundance and matched one of the expected products of isoprene oxidation. HDX results predicted four protogenic groups, which fits one of the isoprene oxidation products observed in the smog chamber.^75^ It corroborates with the rich vegetation of the Rambouillet Forest emitting isoprene in the atmosphere. Moreover, the theoretical hydrolysis product, C_5_H_12_O_4_, was also found in the forest OA with the same number of labile protons matching the stoichiometry with [SO_3_] as a leaving group. Hence, these components may represent a chemical pair, and their association via calculating KMD series could be correct, but this is not a general case. HDX-assisted HRMS enabled a rule out of a portion of precursor/product pairs, leaving only those that fit the stoichiometry of the corresponding chemical reaction. It should be stated that agreement between reaction stoichiometry and HDX results still leaves a level of uncertainty, but it may serve to point out molecules of interest for target analysis, for example, by means of accurate tandem mass spectrometry. In addition to chemical comparison of components, application of HDX may serve to constrain HRMS-based models for physicochemical properties and reactivity of OA^76,77^ and other environmental mixtures^78^ by excluding compounds with the same elemental composition yet different numbers of labile protons.

## Supporting Information Available

Extraction procedure of forest, urban, and marine organic aerosol samples; comparison of water and methanol extracts from urban OA; combination of filters with marine OA according to metal content and their designation; list of AI_con_-based formula classes with atomic constraints; number of lipid formulae containing only alcohol or also carboxyl groups from HDX; number of tentative S-containing groups in CHOS components with <6 oxygen atoms from HDX; general description and visualization of HRMS results for samples under study; impact of (NH_4_)_2_SO_4_ on the efficiency of HDX; visualization of HDX results for individual molecular formulae classes; KMD-series for CHO and CHOS classes in three aerosol types; KMD[O] diagrams for CHO and CHOS formulae in three aerosol types with color-coded number of labile protons; and mass distribution of supported and disproved nitrate hydrolysis pairs in urban OA based on HDX results (PDF)

## References

[ref1] Kanakidou M.; Seinfeld J. H.; Pandis S. N.; Barnes I.; Dentener F. J.; Facchini M. C.; Van Dingenen R.; Ervens B.; Nenes A.; Nielsen C. J.; Swietlicki E.; Putaud J. P.; Balkanski Y.; Fuzzi S.; Horth J.; Moortgat G. K.; Winterhalter R.; Myhre C. E. L.; Tsigaridis K.; Vignati E.; Stephanou E. G.; Wilson J. Organic Aerosol and Global Climate Modelling: A Review. Atmos Chem. Phys. 2005, 5 (4), 1053–1123. 10.5194/acp-5-1053-2005.

[ref2] Shahpoury P.; Zhang Z. W.; Arangio A.; Celo V.; Dabek-Zlotorzynska E.; Harner T.; Nenes A. The Influence of Chemical Composition, Aerosol Acidity, and Metal Dissolution on the Oxidative Potential of Fine Particulate Matter and Redox Potential of the Lung Lining Fluid. Environ. Int. 2021, 148, 106343 10.1016/j.envint.2020.106343. 33454608 PMC7868889

[ref3] Pöschl U. Atmospheric Aerosols: Composition, Transformation, Climate and Health Effects. Angew. Chem., Int. Ed. 2005, 44 (46), 7520–7540. 10.1002/anie.200501122. 16302183

[ref4] Ramanathan V.; Crutzen P. J.; Kiehl J. T.; Rosenfeld D. Atmosphere: Aerosols, Climate, and the Hydrological Cycle. Science 2001, 294 (5549), 2119–2124. 10.1126/science.1064034. 11739947

[ref5] Nizkorodov S. A.; Laskin J.; Laskin A. Molecular Chemistry of Organic Aerosols through the Application of High Resolution Mass Spectrometry. Phys. Chem. Chem. Phys. 2011, 13 (9), 3612–3629. 10.1039/c0cp02032j. 21206953

[ref6] Reemtsma T.; These A.; Venkatachari P.; Xia X.; Hopke P. K.; Springer A.; Linscheid M. Identification of Fulvic Acids and Sulfated and Nitrated Analogues in Atmospheric Aerosol by Electrospray Ionization Fourier Transform Ion Cyclotron Resonance Mass Spectrometry. Anal. Chem. 2006, 78 (24), 8299–8304. 10.1021/ac061320p. 17165819

[ref7] Schmitt-Kopplin P.; Gelencsér A.; Dabek-Zlotorzynska E.; Kiss G.; Hertkorn N.; Harir M.; Hong Y.; Gebefügi I. Analysis of the Unresolved Organic Fraction in Atmospheric Aerosols with Ultrahigh-Resolution Mass Spectrometry and Nuclear Magnetic Resonance Spectroscopy: Organosulfates as Photochemical Smog Constituents. Anal. Chem. 2010, 82 (19), 8017–8026. 10.1021/ac101444r. 20879800

[ref8] Laskin A.; Smith J. S.; Laskin J. Molecular Characterization of Nitrogen-Containing Organic Compounds in Biomass Burning Aerosols Using High-Resolution Mass Spectrometry. Environ. Sci. Technol. 2009, 43 (10), 3764–3771. 10.1021/es803456n. 19544885

[ref9] Bozzetti C.; Sosedova Y.; Xiao M.; Daellenbach K. R.; Ulevicius V.; Dudoitis V.; Mordas G.; Byčenkiene S.; Plauškaite K.; Vlachou A.; Golly B.; Chazeau B.; Besombes J. L.; Baltensperger U.; Jaffrezo J. L.; Slowik J. G.; El Haddad I.; Prévôt A. S. H. Argon Offline-AMS Source Apportionment of Organic Aerosol over Yearly Cycles for an Urban, Rural, and Marine Site in Northern Europe. Atmos Chem. Phys. 2017, 17 (1), 117–141. 10.5194/acp-17-117-2017.

[ref10] Altieri K. E.; Fawcett S. E.; Peters A. J.; Sigman D. M.; Hastings M. G. Marine Biogenic Source of Atmospheric Organic Nitrogen in the Subtropical North Atlantic. Proc. Natl. Acad. Sci. U. S. A. 2016, 113 (4), 925–930. 10.1073/pnas.1516847113. 26739561 PMC4743774

[ref11] Wolf R.; El Haddad I.; Crippa M.; Decesari S.; Slowik J. G.; Poulain L.; Gilardoni S.; Rinaldi M.; Carbone S.; Canonaco F.; Huang R. J.; Baltensperger U.; Prévôt A. S. H. Marine and Urban Influences on Summertime PM2.5 Aerosol in the Po Basin Using Mobile Measurements. Atmos. Environ. 2015, 120, 447–454. 10.1016/j.atmosenv.2015.09.007.

[ref12] Kourtchev I.; Fuller S. J.; Giorio C.; Healy R. M.; Wilson E.; O’Connor I.; Wenger J. C.; McLeod M.; Aalto J.; Ruuskanen T. M.; Maenhaut W.; Jones R.; Venables D. S.; Sodeau J. R.; Kulmala M.; Kalberer M. Molecular Composition of Biogenic Secondary Organic Aerosols Using Ultrahigh-Resolution Mass Spectrometry: Comparing Laboratory and Field Studies. Atmos Chem. Phys. 2014, 14 (4), 2155–2167. 10.5194/acp-14-2155-2014.

[ref13] Riva M.; Da Silva Barbosa T.; Lin Y. H.; Stone A. E.; Gold A.; Surratt J. D. Chemical Characterization of Organosulfates in Secondary Organic Aerosol Derived from the Photooxidation of Alkanes. Atmos Chem. Phys. 2016, 16 (17), 11001–11018. 10.5194/acp-16-11001-2016.

[ref14] Wang K.; Huang R. J.; Brüggemann M.; Zhang Y.; Yang L.; Ni H.; Guo J.; Wang M.; Han J.; Bilde M.; Glasius M.; Hoffmann T. Urban Organic Aerosol Composition in Eastern China Differs from North to South: Molecular Insight from a Liquid Chromatography-Mass Spectrometry (Orbitrap) Study. Atmos Chem. Phys. 2021, 21 (11), 9089–9104. 10.5194/acp-21-9089-2021.

[ref15] Wan Y.; Xing C.; Wang X.; Yang Z.; Huang X.; Ge X.; Du L.; Wang Q.; Yu H. Nontarget Tandem High-Resolution Mass Spectrometry Analysis of Functionalized Organic Compounds in Atmospherically Relevant Samples. Environ. Sci. Technol. Lett. 2022, 9 (12), 1022–1029. 10.1021/acs.estlett.2c00788.

[ref16] Wang K.; Zhang Y.; Huang R. J.; Cao J.; Hoffmann T. UHPLC-Orbitrap Mass Spectrometric Characterization of Organic Aerosol from a Central European City (Mainz, Germany) and a Chinese Megacity (Beijing). Atmos. Environ. 2018, 189, 22–29. 10.1016/j.atmosenv.2018.06.036.

[ref17] Rincón A. G.; Calvo A. I.; Dietzel M.; Kalberer M. Seasonal Differences of Urban Organic Aerosol Composition – an Ultra-High Resolution Mass Spectrometry Study. Environmental Chemistry 2012, 9 (3), 298–319. 10.1071/EN12016.

[ref18] Kourtchev I.; Fuller S.; Aalto J.; Ruuskanen T. M.; McLeod M. W.; Maenhaut W.; Jones R.; Kulmala M.; Kalberer M. Molecular Composition of Boreal Forest Aerosol from Hyytiälä, Finland, Using Ultrahigh Resolution Mass Spectrometry. Environ. Sci. Technol. 2013, 47 (9), 4069–4079. 10.1021/es3051636. 23469832

[ref19] Steimer S. S.; Patton D. J.; Vu T. V.; Panagi M.; Monks P. S.; Harrison R. M.; Fleming Z. L.; Shi Z.; Kalberer M. Differences in the Composition of Organic Aerosols between Winter and Summer in Beijing: A Study by Direct-Infusion Ultrahigh-Resolution Mass Spectrometry. Atmos Chem. Phys. 2020, 20 (21), 13303–13318. 10.5194/acp-20-13303-2020.

[ref20] Kourtchev I.; O’Connor I. P.; Giorio C.; Fuller S. J.; Kristensen K.; Maenhaut W.; Wenger J. C.; Sodeau J. R.; Glasius M.; Kalberer M. Effects of Anthropogenic Emissions on the Molecular Composition of Urban Organic Aerosols: An Ultrahigh Resolution Mass Spectrometry Study. Atmos. Environ. 2014, 89, 525–532. 10.1016/j.atmosenv.2014.02.051.

[ref21] Gurganus S. C.; Wozniak A. S.; Hatcher P. G. Molecular Characteristics of the Water Soluble Organic Matter in Size-Fractionated Aerosols Collected over the North Atlantic Ocean. Mar Chem. 2015, 170, 37–48. 10.1016/j.marchem.2015.01.007.

[ref22] Yassine M. M.; Harir M.; Dabek-Zlotorzynska E.; Schmitt-Kopplin P. Structural Characterization of Organic Aerosol Using Fourier Transform Ion Cyclotron Resonance Mass Spectrometry: Aromaticity Equivalent Approach. Rapid Commun. Mass Spectrom. 2014, 28 (22), 2445–2454. 10.1002/rcm.7038. 25303473

[ref23] Kroll J. H.; Donahue N. M.; Jimenez J. L.; Kessler S. H.; Canagaratna M. R.; Wilson K. R.; Altieri K. E.; Mazzoleni L. R.; Wozniak A. S.; Bluhm H.; Mysak E. R.; Smith J. D.; Kolb C. E.; Worsnop D. R. Carbon Oxidation State as a Metric for Describing the Chemistry of Atmospheric Organic Aerosol. Nat. Chem. 2011, 3 (2), 133–139. 10.1038/nchem.948. 21258386

[ref24] Heald C. L.; Kroll J. H.; Jimenez J. L.; Docherty K. S.; Decarlo P. F.; Aiken A. C.; Chen Q.; Martin S. T.; Farmer D. K.; Artaxo P. A Simplified Description of the Evolution of Organic Aerosol Composition in the Atmosphere. Geophys. Res. Lett. 2010, 37 (8), L08803 10.1029/2010GL042737.

[ref25] Schneider E.; Rüger C. P.; Chacón-Patiño M. L.; Somero M.; Ruppel M. M.; Ihalainen M.; Köster K.; Sippula O.; Czech H.; Zimmermann R. The Complex Composition of Organic Aerosols Emitted during Burning Varies between Arctic and Boreal Peat. Commun. Earth Environ. 2024, 5 (1), 137 10.1038/s43247-024-01304-y.

[ref26] Verkh Y.; Rozman M.; Petrovic M. A Non-Targeted High-Resolution Mass Spectrometry Data Analysis of Dissolved Organic Matter in Wastewater Treatment. Chemosphere 2018, 200, 397–404. 10.1016/j.chemosphere.2018.02.095. 29499520

[ref27] DiDonato N.; Chen H.; Waggoner D.; Hatcher P. G. Potential Origin and Formation for Molecular Components of Humic Acids in Soils. Geochim. Cosmochim. Acta 2016, 178, 210–222. 10.1016/j.gca.2016.01.013.

[ref28] He X.; Huang X. H.; Ma Y.; Huang C.; Yu J. Z. Unambiguous Analysis and Systematic Mapping of Oxygenated Aromatic Compounds in Atmospheric Aerosols Using Ultrahigh-Resolution Mass Spectrometry. Anal. Chem. 2024, 96 (5), 1880–1889. 10.1021/acs.analchem.3c03760. 38253570

[ref29] Spranger T.; Pinxteren D. Van; Reemtsma T.; Lechtenfeld O. J.; Herrmann H. 2D Liquid Chromatographic Fractionation with Ultra-High Resolution MS Analysis Resolves a Vast Molecular Diversity of Tropospheric Particle Organics. Environ. Sci. Technol. 2019, 53 (19), 11353–11363. 10.1021/acs.est.9b03839. 31478645

[ref30] Longnecker K.; Kujawinski E. B. Using Network Analysis to Discern Compositional Patterns in Ultrahigh-Resolution Mass Spectrometry Data of Dissolved Organic Matter. Rapid Commun. Mass Spectrom. 2016, 30 (22), 2388–2394. 10.1002/rcm.7719. 27524402

[ref31] Tziotis D.; Hertkorn N.; Schmitt-Kopplin Ph. Kendrick-Analogous Network Visualisation of Ion Cyclotron Resonance Fourier Transform Mass Spectra: Improved Options for the Assignment of Elemental Compositions and the Classification of Organic Molecular Complexity. Eur. J. Mass Spectrom 2011, 17 (4), 415–421. 10.1255/ejms.1135. 22006638

[ref32] Schymanski E. L.; Jeon J.; Gulde R.; Fenner K.; Ruff M.; Singer H. P.; Hollender J. Identifying Small Molecules via High Resolution Mass Spectrometry: Communicating Confidence. Environ. Sci. Technol. 2014, 48, 2097–2098. 10.1021/es5002105. 24476540

[ref33] Zherebker A.; Kostyukevich Y.; Kononikhin A.; Kharybin O.; Konstantinov A. I.; Zaitsev K. V.; Nikolaev E.; Perminova I. V. Enumeration of Carboxyl Groups Carried on Individual Components of Humic Systems Using Deuteromethylation and Fourier Transform Mass Spectrometry. Anal Bioanal Chem. 2017, 409 (9), 2477–2488. 10.1007/s00216-017-0197-x. 28138744

[ref34] Zherebker A.; Rukhovich G. D.; Sarycheva A.; Lechtenfeld O. J.; Nikolaev E. N. Aromaticity Index with Improved Estimation of Carboxyl Group Contribution for Biogeochemical Studies. Environ. Sci. Technol. 2022, 56 (4), 2729–2737. 10.1021/acs.est.1c04575. 35084826

[ref35] Sareen N.; Carlton A. G.; Surratt J. D.; Gold A.; Lee B.; Lopez-Hilfiker F. D.; Mohr C.; Thornton J. A.; Zhang Z.; Lim Y. B.; Turpin B. J. Identifying Precursors and Aqueous Organic Aerosol Formation Pathways during the SOAS Campaign. Atmos Chem. Phys. 2016, 16 (22), 14409–14420. 10.5194/acp-16-14409-2016.

[ref36] Witt M.; Fuchser J.; Koch B. P. Fragmentation Studies of Fulvic Acids Using Collision Induced Dissociation Fourier Transform Ion Cyclotron Resonance Mass Spectrometry. Anal. Chem. 2009, 81 (7), 2688–2694. 10.1021/ac802624s. 19331432

[ref37] Bürki C.; Reggente M.; Dillner A. M.; Hand J. L.; Shaw S. L.; Takahama S. Analysis of Functional Groups in Atmospheric Aerosols by Infrared Spectroscopy: Method Development for Probabilistic Modeling of Organic Carbon and Organic Matter Concentrations. Atmos. Meas. Tech. 2020, 13 (3), 1517–1538. 10.5194/amt-13-1517-2020.

[ref38] Xia K.; Mei S. S.; Liu C. C.; Liu H.; Yuan R.; Liu S. Characterization of the Organic Functional Group Composition and Sources of Summertime Aerosols in an Eastern City of China. Atmos. Environ. 2022, 277, 119078 10.1016/j.atmosenv.2022.119078.

[ref39] Perminova I. V.; Shirshin E. A.; Konstantinov A. I.; Zherebker A.; Lebedev V. A.; Dubinenkov I. V.; Kulikova N. A.; Nikolaev E. N.; Bulygina E.; Holmes R. M. The Structural Arrangement and Relative Abundance of Aliphatic Units May Effect Long-Wave Absorbance of Natural Organic Matter as Revealed by 1 H NMR Spectroscopy. Environ. Sci. Technol. 2018, 52 (21), 12526–12537. 10.1021/acs.est.8b01029. 30296078

[ref40] Kostyukevich Y.; Kononikhin A.; Popov I.; Nikolaev E. Simple Atmospheric Hydrogen/Deuterium Exchange Method for Enumeration of Labile Hydrogens by Electrospray Ionization Mass Spectrometry. Anal. Chem. 2013, 85 (11), 5330–5334. 10.1021/ac4006606. 23647106

[ref41] Kostyukevich Y.; Acter T.; Zherebker A.; Ahmed A.; Kim S.; Nikolaev E. Hydrogen/Deuterium Exchange in Mass Spectrometry. Mass Spectrom Rev. 2018, 37 (6), 811–853. 10.1002/mas.21565. 29603316

[ref42] Giorio C.; D’Aronco S.; Di Marco V.; Badocco D.; Battaglia F.; Soldà L.; Pastore P.; Tapparo A. Emerging Investigator Series: Aqueous-Phase Processing of Atmospheric Aerosol Influences Dissolution Kinetics of Metal Ions in an Urban Background Site in the Po Valley. Environ. Sci. Process Impacts 2022, 24 (6), 884–897. 10.1039/D2EM00023G. 35611976

[ref43] Pereira D. L.; Gratien A.; Giorio C.; Noyalet G.; Chevaillier S.; Bertin T.; Mebold E.; Cantrell C.; Michoud V.; Di Biagio C.; Picquet-Varrault B.; Hawkins L.; Favez O.; Garret O.; Pronovost D.; Di Antonio L.; de Brito J. F.; Riffault V.; Yu C.; Formenti P. Addressing the Chemical Composition of Secondary Organic Aerosol in the Rural/ Urban Paris Area. EGU General Assembly 2023, EGU23-493 10.5194/egusphere-egu23-493.

[ref44] de Brito J. F.; Formenti P.; Dusanter S.; Jamar M.; Tomas A.; Alleman L.; Perdrix E.; Espina P.; Riffault V.; Yu C.; Di Biagio C.; Gratien A.; Di Antonio L.; Hawkins L.; D’Anna B.; Kammer J.; Monod A.; Petit J.-E.; Deshmukh S.; Poulain L.; Contrasting Aerosol Composition in and out of Paris Plume during the ACROSS Campaign at the Rambouillet Supersite. EGU General Assembly 2023, EGU23-17306 10.5194/egusphere-egu23-17306.

[ref45] Formenti P.; D’Anna B.; Flamant C.; Mallet M.; Piketh S. J.; Schepanski K.; Waquet F.; Auriol F.; Brogniez G.; Burnet F.; Chaboureau J. P.; Chauvigné A.; Chazette P.; Denjean C.; Desboeufs K.; Doussin J. F.; Elguindi N.; Feuerstein S.; Gaetani M.; Giorio C.; Klopper D.; Mallet M. D.; Nabat P.; Monod A.; Solmon F.; Namwoonde A.; Chikwililwa C.; Mushi R.; Welton E. J.; Holben B. The Aerosols, Radiation and Clouds in Southern Africa Field Campaign in Namibia: Overview, Illustrative Observations, and Way Forward. Bull. Am. Meteorol Soc. 2019, 100 (7), 1277–1298. 10.1175/BAMS-D-17-0278.1.

[ref46] Ranninger C.; Schmidt L. E.; Rurik M.; Limonciel A.; Jennings P.; Kohlbacher O.; Huber C. G. Improving Global Feature Detectabilities through Scan Range Splitting for Untargeted Metabolomics by High-Performance Liquid Chromatography-Orbitrap Mass Spectrometry. Anal. Chim. Acta 2016, 930, 13–22. 10.1016/j.aca.2016.05.017. 27265900

[ref47] Kostyukevich Y.; Kononikhin A.; Zherebker A.; Popov I.; Perminova I.; Nikolaev E. Enumeration of Non-Labile Oxygen Atoms in Dissolved Organic Matter by Use of ^16^O/ ^18^O Exchange and Fourier Transform Ion-Cyclotron Resonance Mass Spectrometry. Anal Bioanal Chem. 2014, 406 (26), 6655–6664. 10.1007/s00216-014-8097-9. 25216963

[ref48] Kostyukevich Y.; Kononikhin A.; Popov I.; Kharybin O.; Perminova I.; Konstantinov A.; Nikolaev E. Enumeration of Labile Hydrogens in Natural Organic Matter by Use of Hydrogen/Deuterium Exchange Fourier Transform Ion Cyclotron Resonance Mass Spectrometry. Anal. Chem. 2013, 85 (22), 11007–11013. 10.1021/ac402609x. 24098913

[ref49] Volikov A.; Rukhovich G.; Perminova I. V. NOMspectra: An Open-Source Python Package for Processing High Resolution Mass Spectrometry Data on Natural Organic Matter. J. Am. Soc. Mass Spectrom. 2023, 34 (7), 1524–1527. 10.1021/jasms.3c00003. 37314949

[ref50] Zielinski A. T.; Kourtchev I.; Bortolini C.; Fuller S. J.; Giorio C.; Popoola O. A. M.; Bogialli S.; Tapparo A.; Jones R. L.; Kalberer M. A New Processing Scheme for Ultra-High Resolution Direct Infusion Mass Spectrometry Data. Atmos. Environ. 2018, 178, 129–139. 10.1016/j.atmosenv.2018.01.034.

[ref51] Kozhinov A. N.; Zhurov K. O.; Tsybin Y. O. Iterative Method for Mass Spectra Recalibration via Empirical Estimation of the Mass Calibration Function for Fourier Transform Mass Spectrometry-Based Petroleomics. Anal. Chem. 2013, 85 (13), 6437–6445. 10.1021/ac400972y. 23730691

[ref52] Kellerman A. M.; Guillemette F.; Podgorski D. C.; Aiken G. R.; Butler K. D.; Spencer R. G. M. Unifying Concepts Linking Dissolved Organic Matter Composition to Persistence in Aquatic Ecosystems. Environ. Sci. Technol. 2018, 52 (5), 2538–2548. 10.1021/acs.est.7b05513. 29393627

[ref53] Bianco A.; Riva M.; Baray J. L.; Ribeiro M.; Chaumerliac N.; George C.; Bridoux M.; Deguillaume L. Chemical Characterization of Cloudwater Collected at Puy de Dôme by FT-ICR MS Reveals the Presence of SOA Components. ACS Earth Space Chem. 2019, 3 (10), 2076–2087. 10.1021/acsearthspacechem.9b00153.

[ref54] O’Brien R. E.; Laskin A.; Laskin J.; Rubitschun C. L.; Surratt J. D.; Goldstein A. H. Molecular Characterization of S- and N-Containing Organic Constituents in Ambient Aerosols by Negative Ion Mode High-Resolution Nanospray Desorption Electrospray Ionization Mass Spectrometry: CalNex 2010 Field Study. J. Geophys. Res.: Atmos. 2014, 119 (22), 12706–12720. 10.1002/2014JD021955.

[ref55] Hockaday W. C.; Purcell J. M.; Marshall A. G.; Baldock J. A.; Hatcher P. G. Electrospray and Photoionization Mass Spectrometry for the Characterization of Organic Matter in Natural Waters: A Qualitative Assessment. Limnol Oceanogr 2009, 7, 81–95. 10.4319/lom.2009.7.81.

[ref56] Bressi M.; Sciare J.; Ghersi V.; Mihalopoulos N.; Petit J. E.; Nicolas J. B.; Moukhtar S.; Rosso A.; Féron A.; Bonnaire N.; Poulakis E.; Theodosi C. Sources and Geographical Origins of Fine Aerosols in Paris (France). Atmos Chem. Phys. 2014, 14 (16), 8813–8839. 10.5194/acp-14-8813-2014.

[ref57] Brüggemann M.; Xu R.; Tilgner A.; Kwong K. C.; Mutzel A.; Poon H. Y.; Otto T.; Schaefer T.; Poulain L.; Chan M. N.; Herrmann H. Organosulfates in Ambient Aerosol: State of Knowledge and Future Research Directions on Formation, Abundance, Fate, and Importance. Environ. Sci. Technol. 2020, 54 (7), 3767–3782. 10.1021/acs.est.9b06751. 32157872

[ref58] Zhang J.; Jiang B.; Wang Z.; Liang Y.; Zhang Y.; Xu C.; Shi Q. Molecular Characterisation of Ambient Aerosols by Sequential Solvent Extractions and High-Resolution Mass Spectrometry. Environmental Chemistry 2018, 15 (3), 150–161. 10.1071/EN17197.

[ref59] Xie Q.; Su S.; Chen S.; Zhang Q.; Yue S.; Zhao W.; Du H.; Ren H.; Wei L.; Cao D.; Xu Y.; Sun Y.; Wang Z.; Fu P. Molecular Characterization of Size-Segregated Organic Aerosols in the Urban Boundary Layer in Wintertime Beijing by FT-ICR MS. Faraday Discuss. 2021, 226, 457–478. 10.1039/D0FD00084A. 33237085

[ref60] Mahilang M.; Deb M. K.; Pervez S. Biogenic Secondary Organic Aerosols: A Review on Formation Mechanism, Analytical Challenges and Environmental Impacts. Chemosphere. 2021, 262, 127771 10.1016/j.chemosphere.2020.127771. 32799139

[ref61] Barreira L. M. F.; Ylisirniö A.; Pullinen I.; Buchholz A.; Li Z.; Lipp H.; Junninen H.; Hõrrak U.; Noe S. M.; Krasnova A.; Krasnov D.; Kask K.; Talts E.; Niinemets Ü.; Ruiz-Jimenez J.; Schobesberger S. The Importance of Sesquiterpene Oxidation Products for Secondary Organic Aerosol Formation in a Springtime Hemiboreal Forest. Atmos Chem. Phys. 2021, 21 (15), 11781–11800. 10.5194/acp-21-11781-2021.

[ref62] Salvador C. M.; Chou C. C. K.; Ho T. T.; Tsai C. Y.; Tsao T. M.; Tsai M. J.; Su T. C. Contribution of Terpenes to Ozone Formation and Secondary Organic Aerosols in a Subtropical Forest Impacted by Urban Pollution. Atmosphere (Basel) 2020, 11 (11), 1232 10.3390/atmos11111232.

[ref63] Zhang Y.; Gao X.; Hou X.; Liu M.; Han J.; Zhang H. Chemical Characterization of Rural Organic Aerosol in the North China Plain Using Ultrahigh-Resolution Mass Spectrometry. Atmosphere (Basel) 2023, 14 (11), 1636 10.3390/atmos14111636.

[ref64] Zherebker A.; Kostyukevich Y.; Kononikhin A.; Roznyatovsky V. A.; Popov I.; Grishin Y. K.; Perminova I. V.; Nikolaev E. High Desolvation Temperature Facilitates the ESI-Source H/D Exchange at Non-Labile Sites of Hydroxybenzoic Acids and Aromatic Amino Acids. Analyst 2016, 141 (8), 2426–2434. 10.1039/C5AN02676H. 27002310

[ref65] Hatvany J. B.; Liyanage O. T.; Gallagher E. S. Effect of PH on In-Electrospray Hydrogen/Deuterium Exchange of Carbohydrates and Peptides. J. Am. Soc. Mass Spectrom. 2024, 35 (3), 441–448. 10.1021/jasms.3c00341. 38323552

[ref66] Gallimore P. J.; Giorio C.; Mahon B. M.; Kalberer M. Online Molecular Characterisation of Organic Aerosols in an Atmospheric Chamber Using Extractive Electrospray Ionisation Mass Spectrometry. Atmos Chem. Phys. 2017, 17 (23), 14485–14500. 10.5194/acp-17-14485-2017.

[ref67] Parshintsev J.; Hyötyläinen T. Methods for Characterization of Organic Compounds in Atmospheric Aerosol Particles. Anal. Bioanal. Chem. 2014, 407 (20), 5877–5897. 10.1007/s00216-014-8394-3. 25542579

[ref68] Pohlabeln A. M.; Dittmar T. Novel Insights into the Molecular Structure of Non-Volatile Marine Dissolved Organic Sulfur. Mar Chem. 2015, 168, 86–94. 10.1016/j.marchem.2014.10.018.

[ref69] Zherebker A.; Kostyukevich Y.; Volkov D. S.; Chumakov R. G.; Friederici L.; Rüger C. P.; Kononikhin A.; Kharybin O.; Korochantsev A.; Zimmermann R.; Perminova I. V.; Nikolaev E. Speciation of Organosulfur Compounds in Carbonaceous Chondrites. Sci. Rep. 2021, 11 (1), 7410 10.1038/s41598-021-86576-6. 33795703 PMC8016918

[ref70] Srivastava D.; Vu T. V.; Tong S.; Shi Z.; Harrison R. M. Formation of Secondary Organic Aerosols from Anthropogenic Precursors in Laboratory Studies. npj Clim. Atmos. Sci. 2022, 5 (1), 22 10.1038/s41612-022-00238-6.

[ref71] Bateman A. P.; Walser M. L.; Desyaterik Y.; Laskin J.; Laskin A.; Nizkorodov S. A. The Effect of Solvent on the Analysis of Secondary Organic Aerosol Using Electrospray Ionization Mass Spectrometry. Environ. Sci. Technol. 2008, 42 (19), 7341–7346. 10.1021/es801226w. 18939568

[ref72] Huang L.; Liu T.; Grassian V. H. Radical-Initiated Formation of Aromatic Organosulfates and Sulfonates in the Aqueous Phase. Environ. Sci. Technol. 2020, 54 (19), 11857–11864. 10.1021/acs.est.0c05644. 32969227

[ref73] Passananti M.; Kong L.; Shang J.; Dupart Y.; Perrier S.; Chen J.; Donaldson D. J.; George C. Organosulfate Formation through the Heterogeneous Reaction of Sulfur Dioxide with Unsaturated Fatty Acids and Long-Chain Alkenes. Angew. Chem., Int. Ed. 2016, 55 (35), 10336–10339. 10.1002/anie.201605266. 27458109

[ref74] Surratt J. D.; Gómez-González Y.; Chan A. W. H.; Vermeylen R.; Shahgholi M.; Kleindienst T. E.; Edney E. O.; Offenberg J. H.; Lewandowski M.; Jaoui M.; Maenhaut W.; Claeys M.; Flagan R. C.; Seinfeld J. H. Organosulfate Formation in Biogenic Secondary Organic Aerosol. J. Phys. Chem. A 2008, 112 (36), 8345–8378. 10.1021/jp802310p. 18710205

[ref75] Nestorowicz K.; Jaoui M.; Jan Rudzinski K.; Lewandowski M.; Kleindienst T. E.; Spólnik G.; Danikiewicz W.; Szmigielski R. Chemical Composition of Isoprene SOA under Acidic and Non-Acidic Conditions: Effect of Relative Humidity. Atmos. Chem. Phys. 2018, 18 (24), 18101–18121. 10.5194/acp-18-18101-2018. 32158471 PMC7063744

[ref76] Tumminello P. R.; James R. C.; Kruse S.; Kawasaki A.; Cooper A.; Guadalupe-Diaz I.; Zepeda K. L.; Crocker D. R.; Mayer K. J.; Sauer J. S.; Lee C.; Prather K. A.; Slade J. H. Evolution of Sea Spray Aerosol Particle Phase State across a Phytoplankton Bloom. ACS Earth Space Chem. 2021, 5 (11), 2995–3007. 10.1021/acsearthspacechem.1c00186.

[ref77] DeRieux W. S. W.; Li Y.; Lin P.; Laskin J.; Laskin A.; Bertram A. K.; Nizkorodov S. A.; Shiraiwa M. Predicting the Glass Transition Temperature and Viscosity of Secondary Organic Material Using Molecular Composition. Atmos Chem. Phys. 2018, 18 (9), 6331–6351. 10.5194/acp-18-6331-2018.

[ref78] Plamper P.; Lechtenfeld O. J.; Herzsprung P.; Groß A. A Temporal Graph Model to Predict Chemical Transformations in Complex Dissolved Organic Matter. Environ. Sci. Technol. 2023, 57 (46), 18116–18126. 10.1021/acs.est.3c00351. 37159837 PMC10666529

