# Supplementary material for: Decreasing the Uncertainty in the Comparison of Molecular Fingerprints of Organic Aerosols with H/D Exchange Mass Spectrometry
Source: Environ Sci Technol. 2024 Nov 8;58(46):20468–79. doi: 10.1021/acs.est.4c06749 (PMC11580170; doi:10.1021/acs.est.4c06749)
Supplement: Supplementary file 1 — es4c06749_si_001.pdf [file es4c06749_si_001.pdf]

Supporting information

Decreasing the uncertainty in the comparison of molecular fingerprints of organic aerosols with H/D exchange mass spectrometry

Alexander Zherebker[1]*, Oliver Babcock[1], Diana L. Pereira[2], Sara D'Aronco[1], Daniele Filippi[1,3], Lidia Soldà[3], Vincent Michoud[2], Aline Gratien[2], Manuela Cirtog[4], Christopher Cantrell[4], Paola Formenti[2], and Chiara Giorio[1]*

[1]Yusuf Hamied Department of Chemistry, University of Cambridge, Cambridge, CB2 1EW, United Kingdom
[2]Université Paris Cité and Univ Paris Est Creteil, CNRS, LISA, Paris, F-75013, France
[3]Department of Chemical Sciences, Università degli Studi di Padova, 35131 Padova, Italy
[4]Université Paris Est Creteil and Université Paris Cité, CNRS, LISA, Créteil, F-94010, France

Corresponding authors contact: az459@cam.ac.uk, cg525@cam.ac.uk

This document contains 10 pages, 13 figures, 4 tables, and additional information on the experimental procedures and data analysis.

Extraction procedure of forest organic aerosol samples.

The extraction procedure was adapted from Kourtchev et al.[1] A third of quartz filters were extracted three times by 30 min sonication in slurry ice with 3 mL of methanol (LC-MS grade, Fisher Scientific) in pre-cleaned glass vials. Extracts were combined for each sample and filtered with 0.45 µm and 0.2 µm syringe filters (Iso-DiscTM Filters PTFE, ∅ 4 mm) consecutively. The solvent was partially evaporated using a gentle stream of nitrogen to 400 µl. The final solutions were stored at -18°C prior to analysis. Before analysis samples were diluted 5 times with water-methanol (1:9) mixture. Designation for forest OA samples consisted of location name Ramb (for Rambouillet), the day of collection (e.g. 03_07 for July 3rd) and the time of the day: D and N for day and night, respectively.

Extraction procedure of urban organic aerosol samples.

Extraction procedure was adapted from the previous works[2,3]. A quarter of Teflon filters were pre-soaked in 8 mL of MilliQ water in pre-cleaned glass vials and further sonicated for 40 minutes in slurry ice. Due to hydrophobicity of PTFE, filters were submerged in solution by plastic stopper. The extract was filtered through 0.2 µm syringe filters (Iso-DiscTM Filters PTFE, ∅ 4 mm). The filtered solutions were acidified to pH 2 with 0.1M HCl followed by solid-phase extraction (SPE) on PPL cartridges. Briefly, the SPE cartridge was activated by passing 1 bed volume of methanol followed by equilibration with 1.5 bed volumes of 0.1 % formic acid in water. The filtered solutions were passed through the cartridge at a speed of approx. 1 drop per second under a gentle N_2 gas pressure required to achieve the required flowrate. The cartridge with sorbed material was washed with 3 volumes of 0.1 % formic acid and then dried fully with N_2 gas for 10 minutes. The organic fraction was eluted with 1 ml methanol. The solvent was partially evaporated using a gentle stream of nitrogen to 200 µl. Before analysis, 20 µl water was added to the methanol eluate. Designation of urban OA samples consisted of the location name Padua, and the internal laboratory number, e.g. T10B.

Extraction procedure of marine organic aerosol samples.

Extraction of marine samples was conducted similarly to urban samples from the quarters of Teflon filters. It was estimated that on average filters contained 0.03 mg of organic carbon (OC, unpublished data). Due to low OC content filters were pulled together based on a preliminary ICP-MS data on the content of Fe and Mn, and IC data on non-sea-salt sulphates (Table S1). The sections of designated filters were extracted consecutively with the same solvent mixture to get the highest organic carbon content before SPE.

Comparison of water and methanol extracts from urban OA.

To perform the comparison a section of the T10B filter sample was extracted by methanol similar to forest OA samples. Mass spectra were acquired at a Q Exactive Orbitrap mass spectrometer (Thermo Scientific™, Bremen, Germany) with the identical ion source and applying the same analytical procedure as for samples analyzed on the LTQ Orbitrap. Figure S6 (A) depicts van Krevelen diagrams for the extracts obtained with methanol and water followed by solid-phase extraction. The visual inspection shows the resemblance between these samples. Yet, it is noticeable that methanol resulted in the higher proportion of highly unsaturated components (Fig. S6B). Taking that negative electrospray is biased toward acidic components, it can be concluded that methanol and water extract similar ionizable components. Therefore, difference in extraction protocol has a scarce impact on the peculiarity of forest OA molecular fingerprint. It is worth mentioning that comparison of molecular fingerprints obtained from different instrumentations is unfavorable due to inherent differences in the ion detection.[1] However, representation of the data using average metrics from van Krevelen diagram (e.g. classes) is considered robust.[2]

Table S1. Combination of filters with marine OA according to metal content and their designation

Element's content	Number of combined filters	Estimated final OC, $mg \cdot L^{-1}$	Sample designation
High Fe, low Mn	4	45	T9T12-14
High Mn, low Fe	5	71	T17T20-23
Low Mn, low Fe, high non-sea-salt sulphate	4	59	T31-33T38
Low Mn, low Fe, high non-sea-salt sulphate	4	22	T26-27T36-37
Low non-sea-salt sulphate, high Mn	5	61	T10T15-16T24-25
High non-sea-salt sulphate, low Mn	4	31	T28T30T34-35
Unclassified	3	48	T3_T5
Unclassified	3	101	T6_T8

* Upper estimation is based on 10 L/min OA collection rate, 25% of filter extracted, 50% SPE efficiency and final volume for analysis of 250 µl

Table S2. The list of AI_{con}-based formulae classes with atomic constraints (adapted from Kellerman et al.[6])

Formulae class	O/C	H/C	AI_{con}
Lipids	< 0.3	≥ 1.5	-
N-containing saturated compounds (N-saturated)	-	≥ 1.5*	-
Aliphatics	≥ 0.3	≥ 1.5**	-
Unsaturated (low O/C)	< 0.5	< 1.5	< 0.5
Unsaturated (high O/C)	≥ 0.5	< 1.5	< 0.5
Aromatic (low O/C)	< 0.5	-	≥ 0.5 and < 0.67
Aromatic (high O/C)	≥ 0.5	-	≥ 0.5 and < 0.67
Condensed (low O/C)	< 0.5	-	≥ 0.67
Condensed (high O/C)	≥ 0.5	-	≥ 0.67

* N > 0; ** N = 0

Table S3. Number of lipid formulae containing only alcohol or also carboxyl groups from HDX

OA sample	Contains OH-only	Can contain COOH
Urban (O > 2)	3	22
Forest (O > 2)	0	94
Marine (O > 2)	0	49

Table S4. Number of tentative S-containing groups in CHOS components with < 6 oxygen atoms from HDX, where R and R_1 represent CHO-only carbon structural fragments

OA sample	$R\text{-}SO_4$	$R\text{-}SO_3$	$R\text{-}SO_2\text{-}R_1$	$R\text{-}SO\text{-}R_1$	R-SH
Urban	40	18	2	6	0
Forest	52	25	7	7	2
Marine	54	38	4	7	9

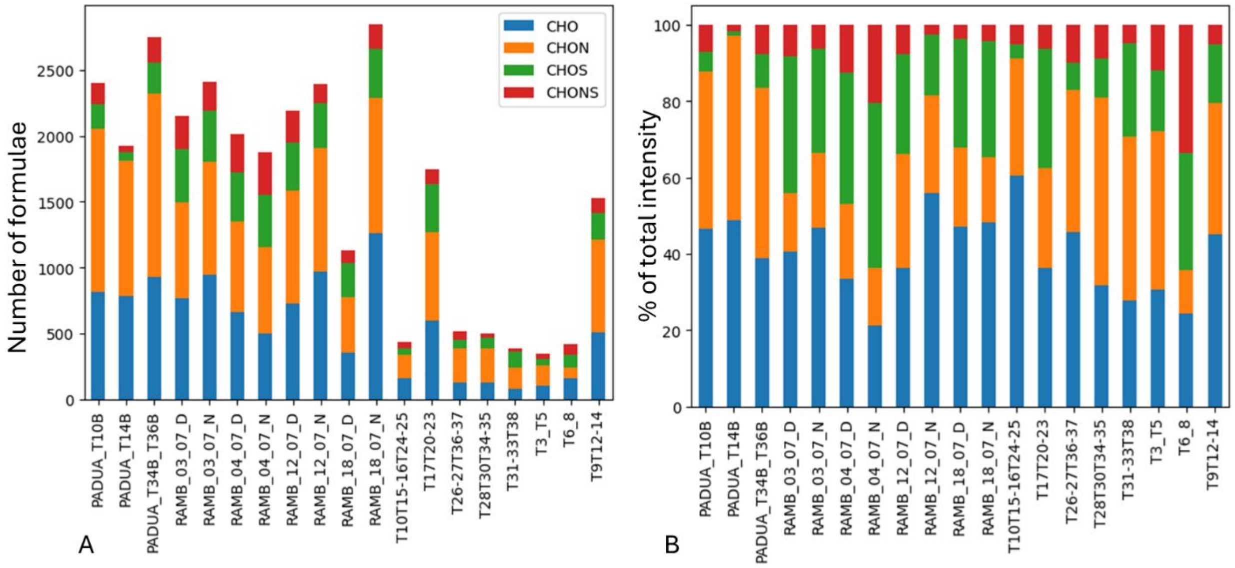

Figure S1. Absolute number (A) and intensity contribution (B) of different $C_cH_hO_oN_nS_s$ formulae in samples under study for the sites: Padua, Rambouillet and Henties Bay.

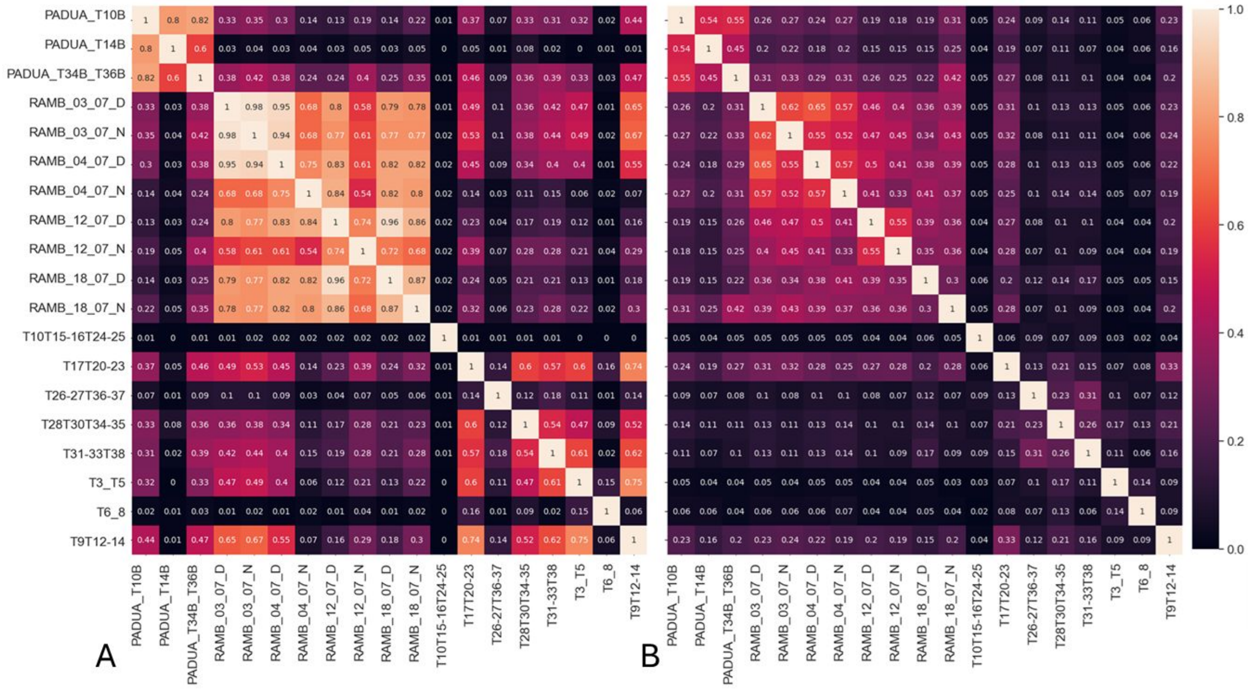

Figure S2. Pairwise comparison of samples under study based on HRMS results: A) cosine similarity, B) Jaccard similarity

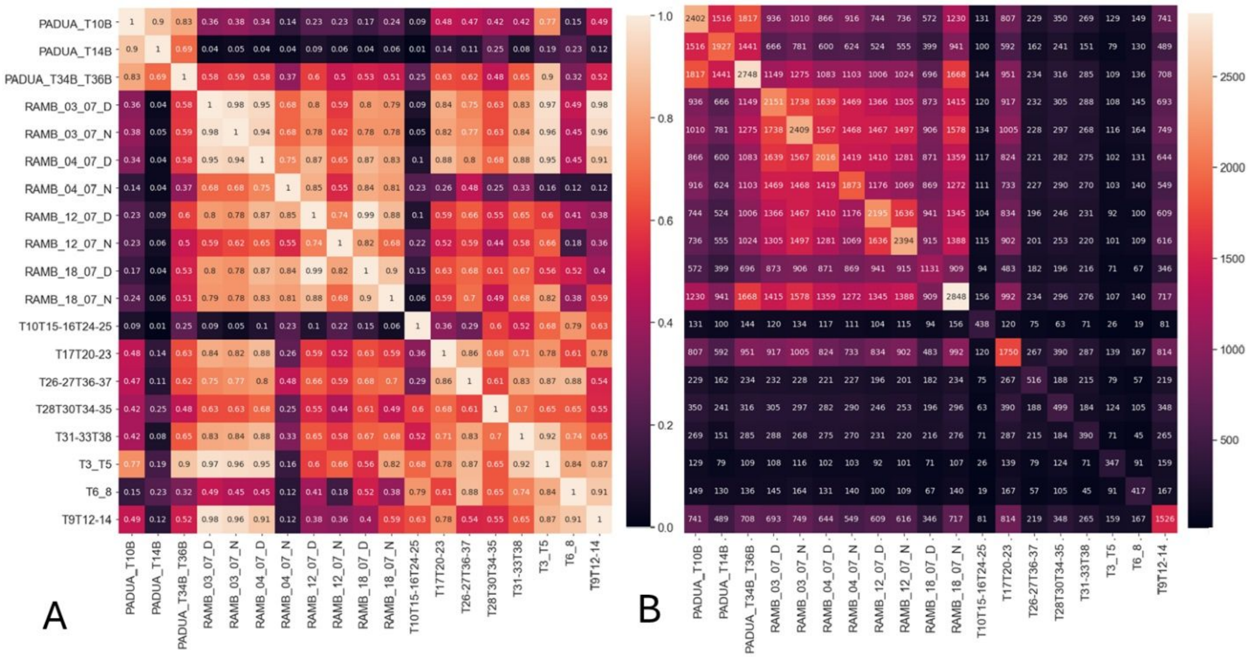

Figure S3. A) Pairwise correlation of intensities of common formulae as well as B) number of intersected formulae

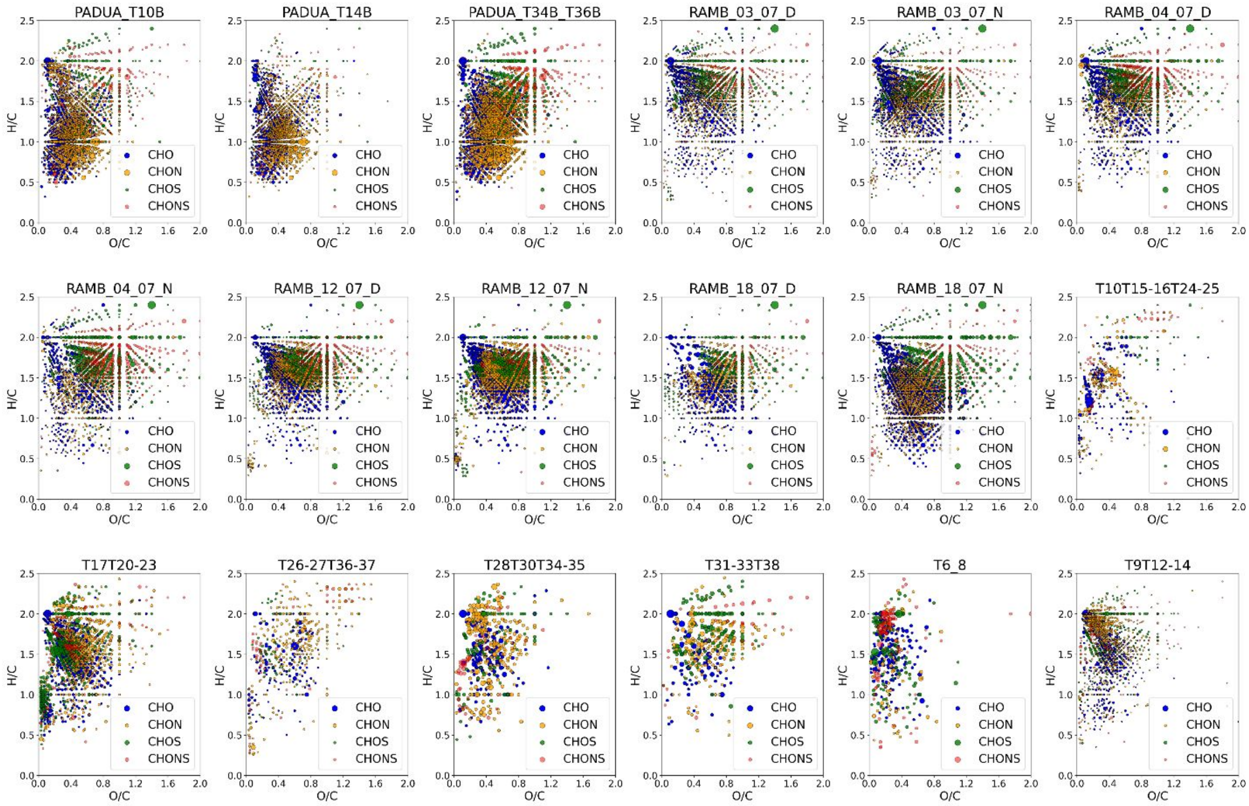

Figure S4. VK diagrams of samples under study based on HRMS data for the sites: Padua, Rambouillet and Henties Bay.

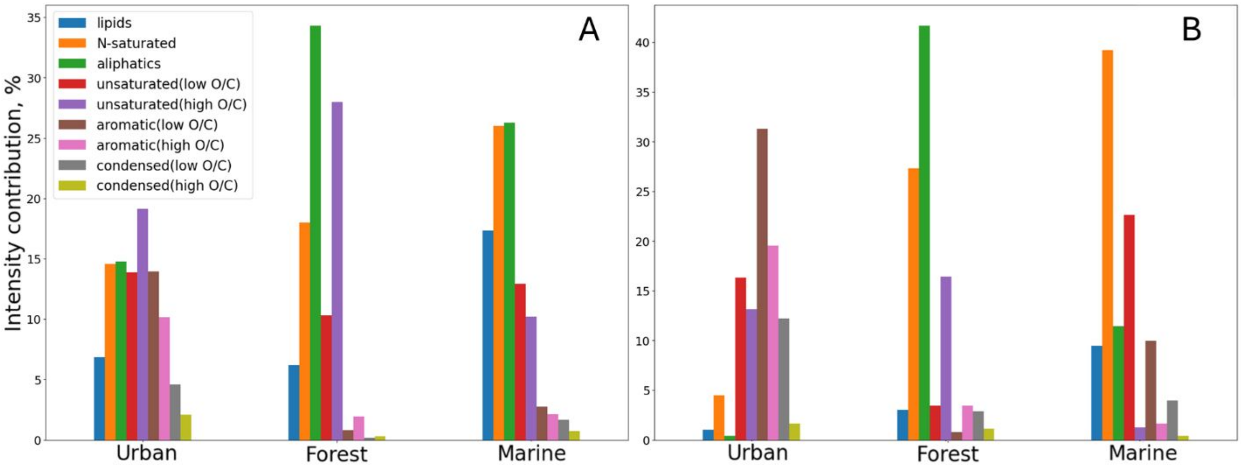

Figure S5. Intensity contribution of AI_{con}-based classes in A) common and B) unique formulae in three aerosol types under study according to Table S2.

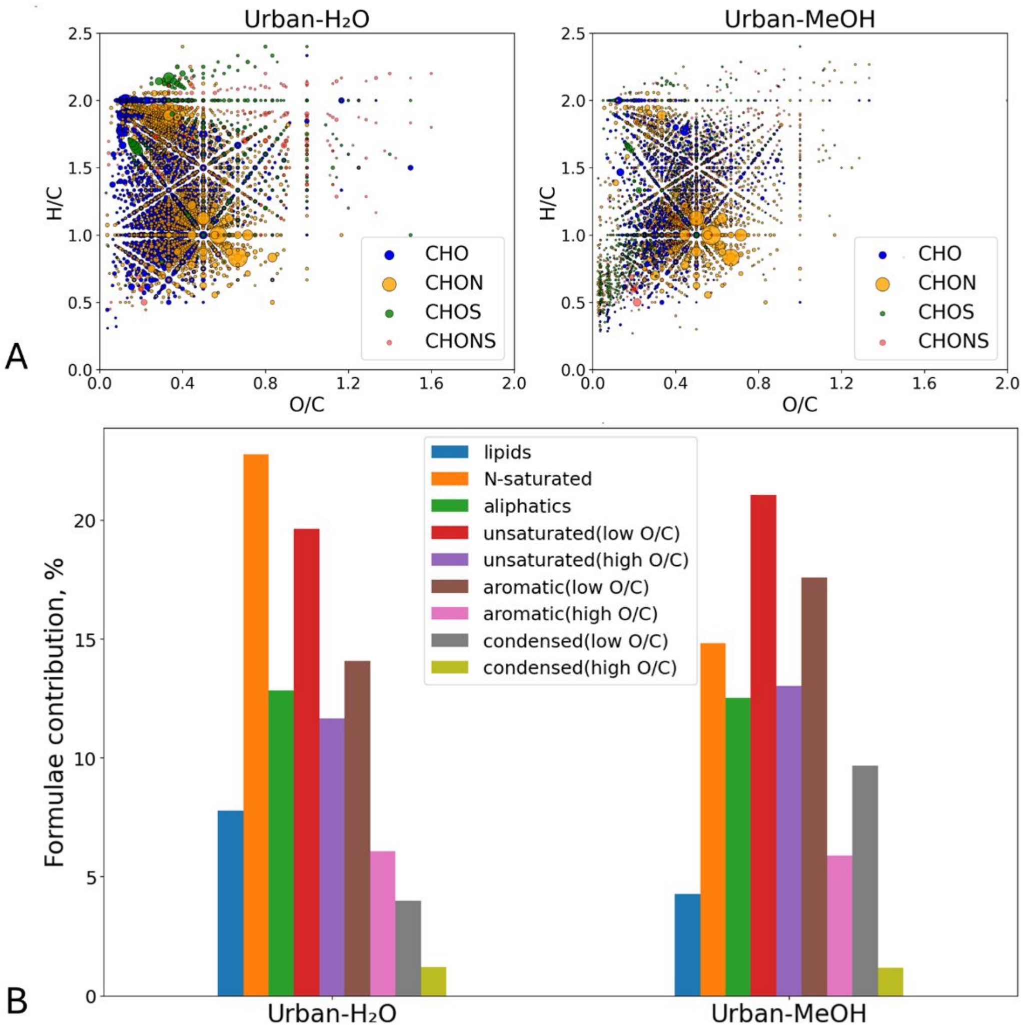

Figure S6. Comparison of water and methanol extracts from urban OA: A) van Krevelen diagram, B) distribution of AI_{con}-based classes

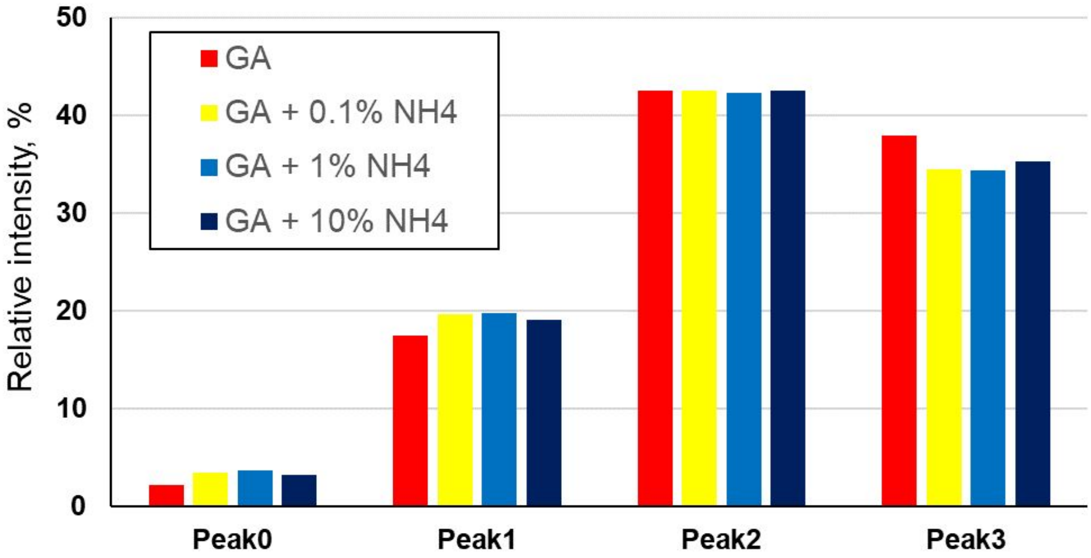

Figure S7. Relative intensities of peaks for parent and labeled ions of gallic acid in HDX HRMS experiment with the addition of three concentrations of $(NH_4)_2SO_4$.

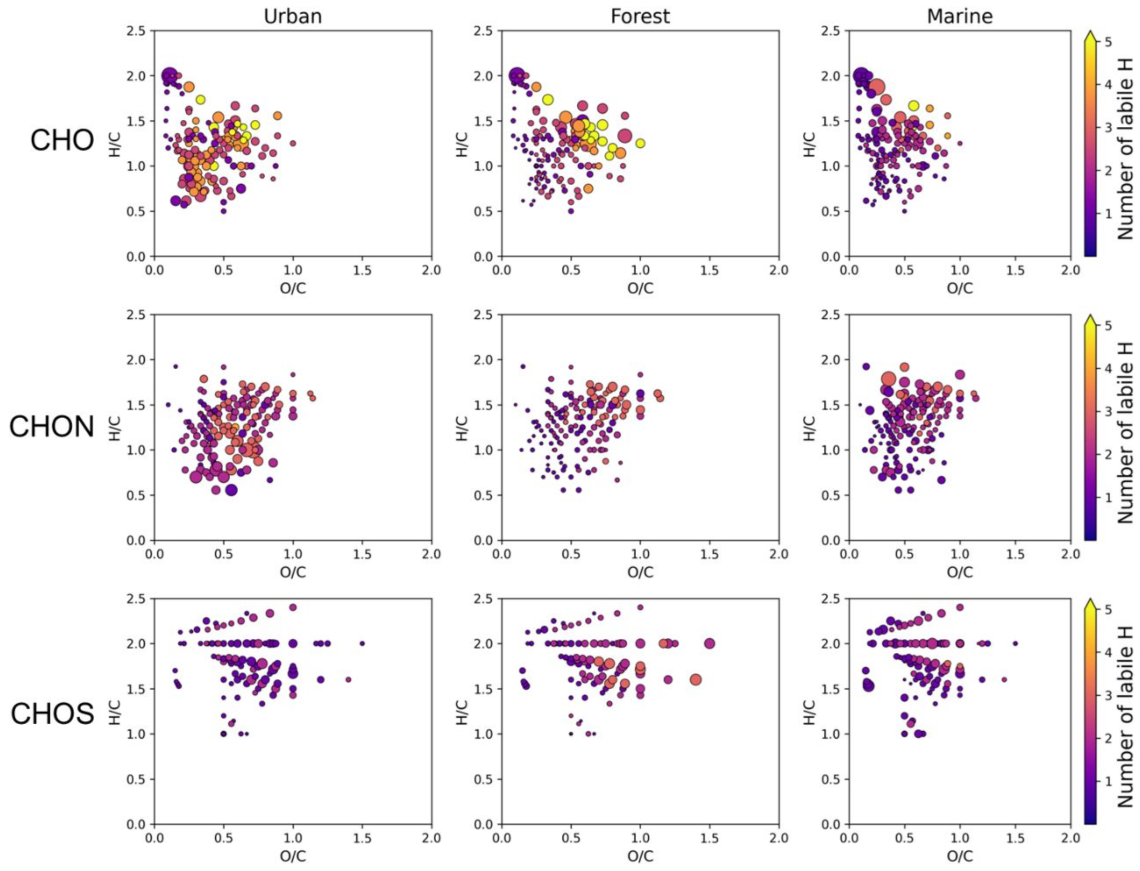

Figure S8. Class-specific distribution of labile protons on VK diagrams for common formulae in three aerosol types under study.

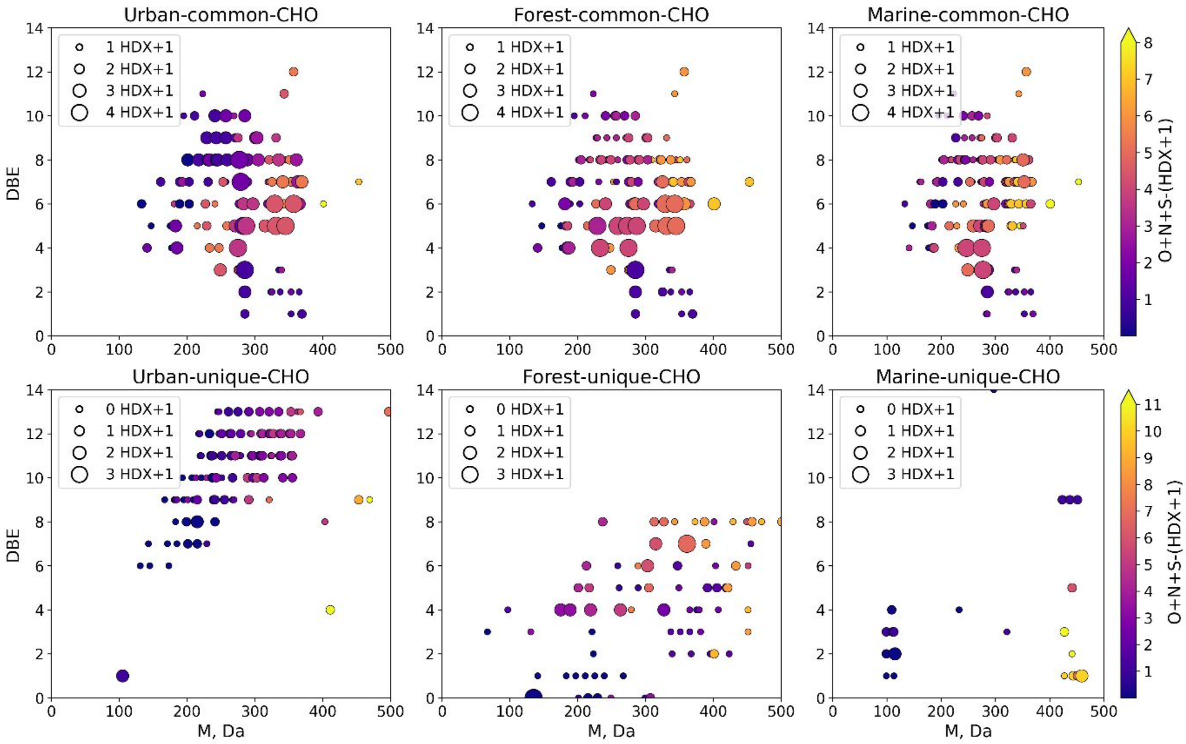

Figure S9. Double bond equivalent (DBE) versus molecular mass plots with size-coded number of labile protons (HDX+1) and the color-coded number of residual heteroatoms (non-protogenic heteroatoms) for common (upper panel) and unique (lower panel) CHO-only molecular features for each OA source

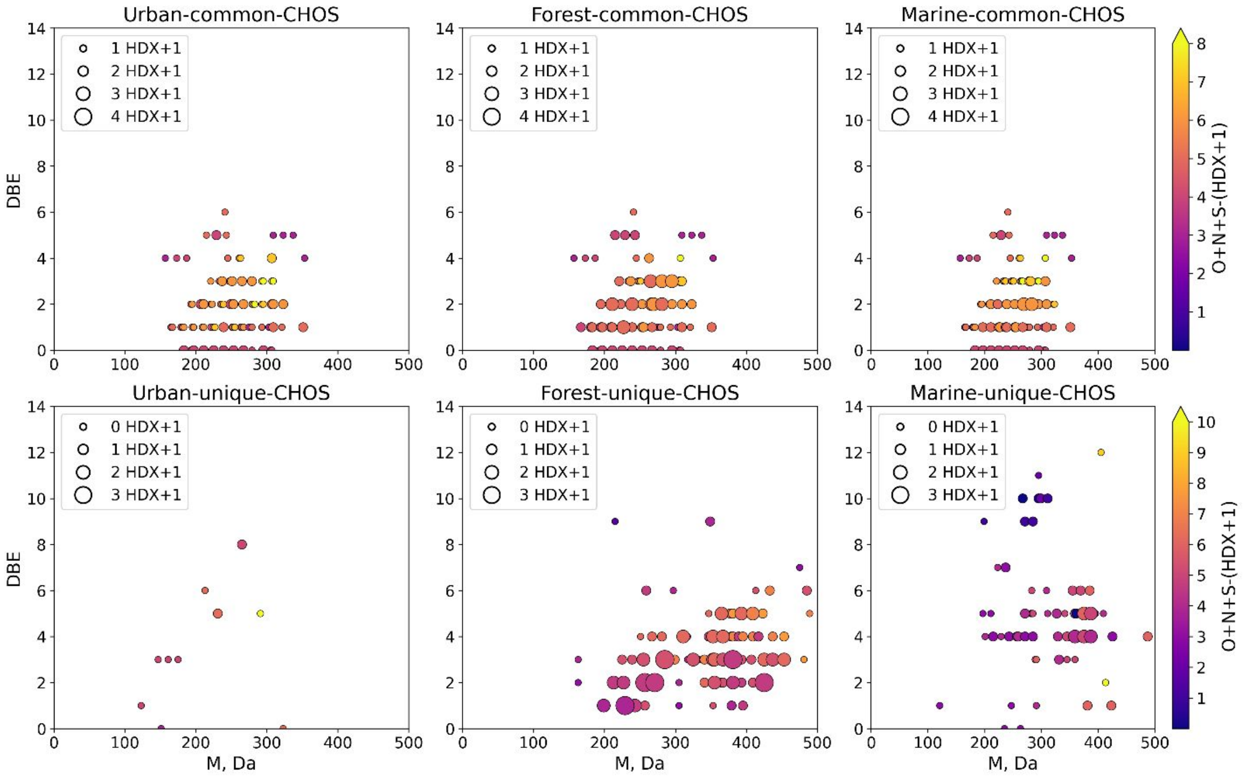

Figure S10. Double bond equivalent (DBE) versus molecular mass plots with size-coded number of labile protons (HDX+1) and the color-coded number of residual heteroatoms (non-protogenic heteroatoms) for common (upper panel) and unique (lower panel) CHOS-only molecular features for each OA source

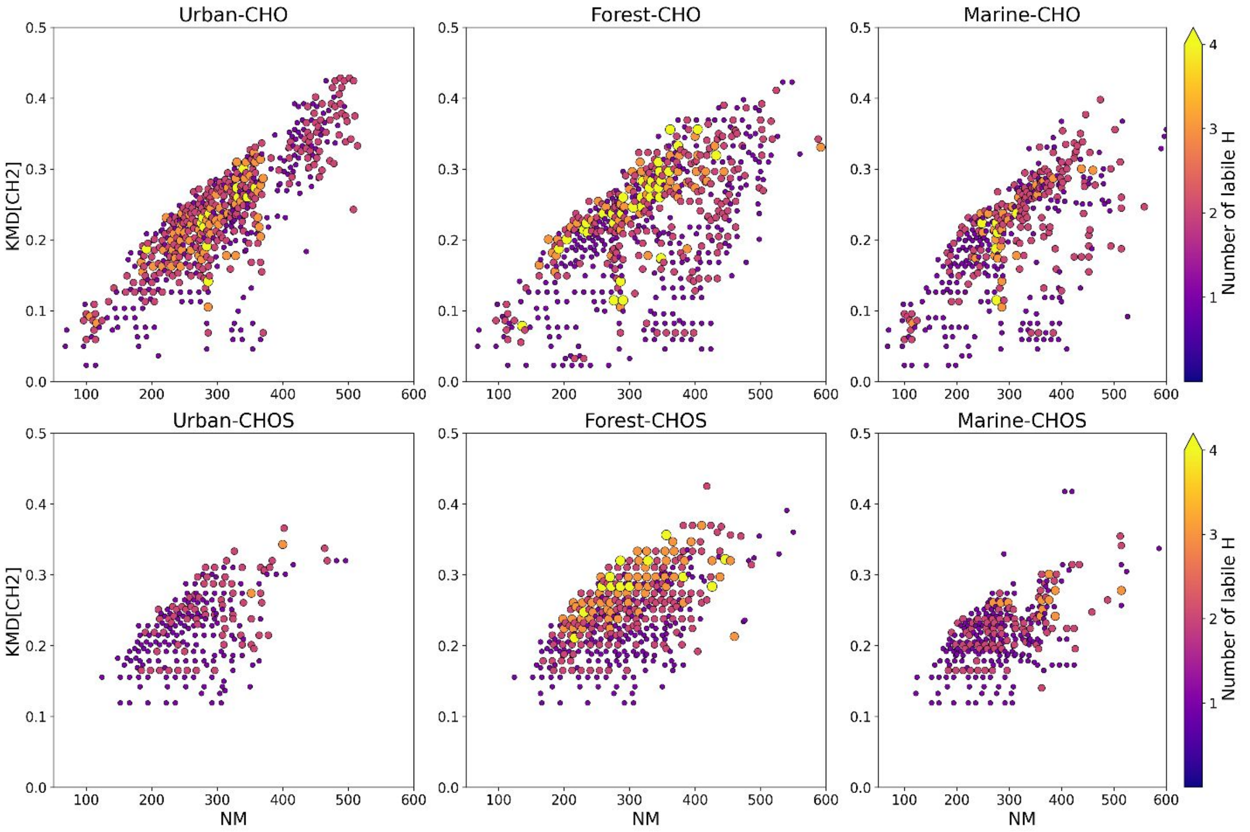

Figure S11. KMD[CH_2] diagrams for CHO and CHOS formulae in three aerosol types with color-coded number of labile protons.

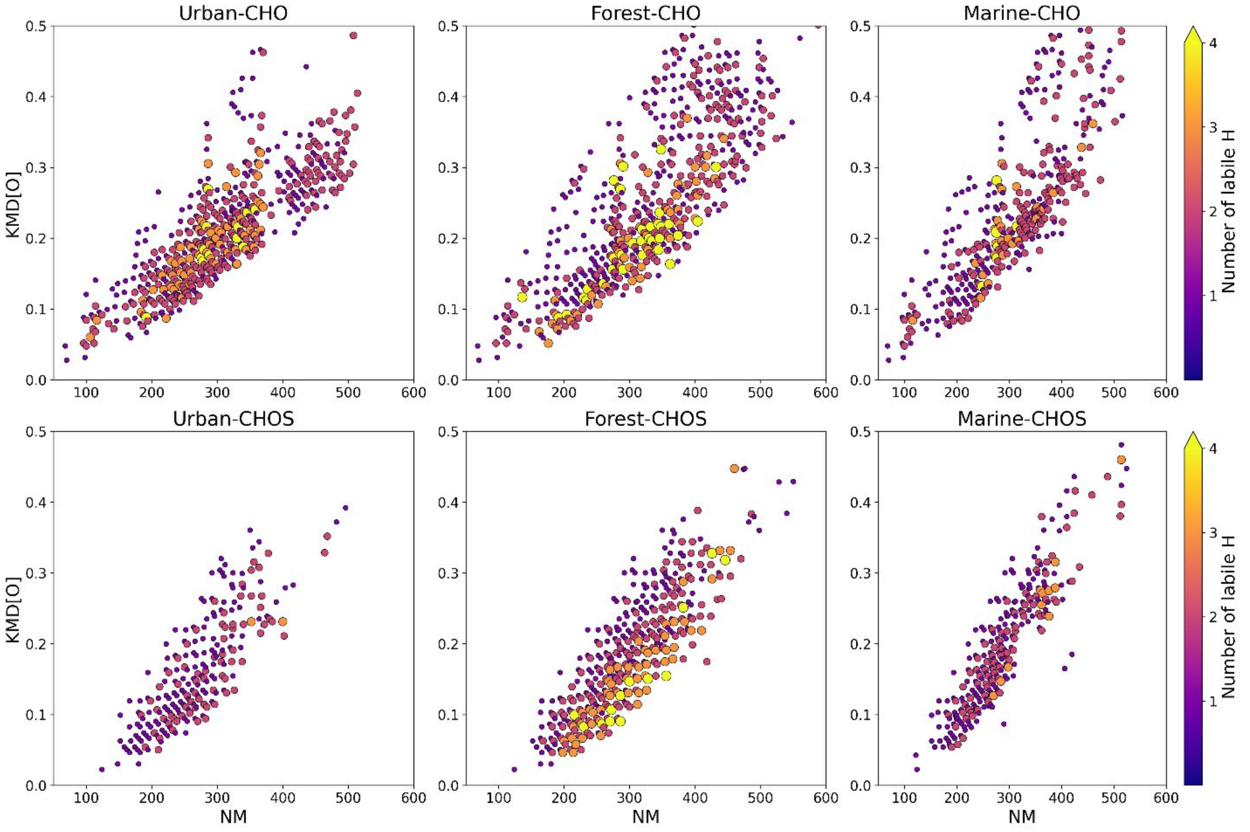

Figure S12. KMD[O] diagrams for CHO and CHOS formulae in three aerosol types with color-coded number of labile protons.

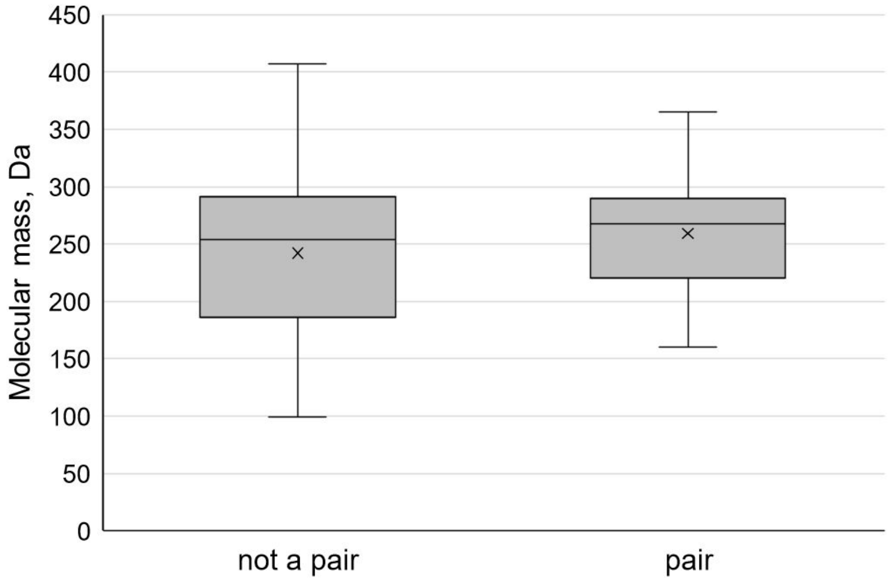

Figure S13. Mass distribution of supported and disproved nitrates hydrolysis pairs in Urban OA based in HDX results.

References

(1) Kourtchev, I.; Fuller, S.; Aalto, J.; Ruuskanen, T. M.; McLeod, M. W.; Maenhaut, W.; Jones, R.; Kulmala, M.; Kalberer, M. Molecular Composition of Boreal Forest Aerosol from Hyytiälä, Finland, Using Ultrahigh Resolution Mass Spectrometry. *Environ Sci Technol* **2013**, *47* (9), 4069–4079.

(2) Rincón, A. G.; Calvo, A. I.; Dietzel, M.; Kalberer, M. Seasonal Differences of Urban Organic Aerosol Composition – an Ultra-High Resolution Mass Spectrometry Study. *Environmental Chemistry* **2012**, *9* (3), 298–319.

(3) Dittmar, T.; Koch, B.; Hertkorn, N.; Kattner, G. A Simple and Efficient Method for the Solid-Phase Extraction of Dissolved Organic Matter (SPE-DOM) from Seawater. *Limnol. Oceanogr. Methods* **2008**, *6*, 230–235.

(4) Hawkes, J. A.; d'Andrilli, J.; Agar, J. N.; Barrow, M. P.; Berg, S. M.; Catalán, N.; Chen, H.; Chu, R. K.; Cole, R. B.; Dittmar, T.; others. An International Laboratory Comparison of Dissolved Organic Matter Composition by High Resolution Mass Spectrometry: Are We Getting the Same Answer? *Limnol Oceanogr Methods* **2020**, *18* (6), 235–258.

(5) Zherebker, A.; Kim, S.; Schmitt-Kopplin, P.; Spencer, R. G. M.; Lechtenfeld, O.; Podgorski, D. C.; Hertkorn, N.; Harir, M.; Nurfajin, N.; Koch, B.; others. Interlaboratory Comparison of Humic Substances Compositional Space as Measured by Fourier Transform Ion Cyclotron Resonance Mass Spectrometry (IUPAC Technical Report). *Pure and Applied Chemistry* **2020**, *92* (9), 1447–1467.

(6) Kellerman, A. M.; Dittmar, T.; Kothawala, D. N.; Tranvik, L. J. Chemodiversity of Dissolved Organic Matter in Lakes Driven by Climate and Hydrology. *Nat Commun* **2014**, *5*, 3804. https://doi.org/10.1038/ncomms4804.
